# Self‐Assembling Glycopeptide Conjugate as a Versatile Platform for Mimicking Complex Polysaccharides

**DOI:** 10.1002/advs.202001264

**Published:** 2020-07-02

**Authors:** Hanxuan Wang, Zhichao Liu, Chuanjing An, Haoting Li, Fanlei Hu, Suwei Dong

**Affiliations:** ^1^ State Key Laboratory of Natural and Biomimetic Drugs and Department of Chemical Biology School of Pharmaceutical Sciences Peking University Beijing 100191 China; ^2^ Department of Rheumatology and Immunology Peking University People's Hospital & Beijing Key Laboratory for Rheumatism Mechanism and Immune Diagnosis (BZ0135) Beijing 100044 China

**Keywords:** glycopeptide conjugates, immune activation, polysaccharides, self‐assembly

## Abstract

Polysaccharides are a class of carbohydrates that play pivotal roles in living systems such as being chemical messengers in many vital biological pathways. However, the complexity and heterogeneity of these natural structures have posed daunting challenges on their production, characterization, evaluation, and applications. While there have been various types of synthetic skeletons that could mimic some biological aspects of polysaccharides, a safer and more easily accessed system is still desired to avoid the unnatural components and difficulties in modifying the structures. In this work, conveniently accessible self‐assembling glycopeptide conjugates are developed, where the natural O‐glycosidic linkages and phosphoryl modifications assist the self‐assembly and concurrently reduce the risk of toxicity. The generated nanoparticles in aqueous solution offer a multivalent display of structurally controllable carbohydrates as mimics of polysaccharides, among which a mannosylated version exhibits immunostimulatory effects in both cellular assays and vaccination of mice. The obtained results demonstrate the potential of this glycopeptide conjugate‐derived platform in exploiting the intriguing properties of carbohydrates in a more structurally maneuverable fashion.

## Graphical abstract

A glycopeptide‐based self‐assembly containing monosaccharides and phosphates is established to display simple carbohydrates in a multivalent format, mimicking natural complex polysaccharides. Among several evaluated glycoforms, the self‐assembled mannosylated nanoparticles exhibit immunostimulatory effects both in vitro and in vivo.

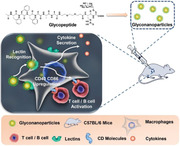

## 1 Introduction

Carbohydrates represent a major class of biomolecules existing in nature that have multifaceted functions across the fields from energy source to biological structures.^[^ ^1^, ^2^ ^]^ A prominent feature of carbohydrates is their extremely diverse and complex structures. For instance, several types of carbohydrates, including glycolipids, glycoproteins, and glycocalyx, have been demonstrated to be able to act as chemical messengers in living systems, such as triggering human immune responses.^[^ ^3^, ^4^ ^]^ Notably, most interactions between these natural carbohydrates and their receptors are required to be multivalent to achieve exponentially increased binding affinity and specificity.^[^ ^5^, ^6^, ^7^ ^]^ A number of natural polysaccharides, including mannan, *α*‐glucan, and *β*‐glucan, can bind to multivalent pattern recognition receptors (PRRs), such as dectins, mannose receptors, dendritic cell‐specific intercellular adhesion molecule‐3‐grabbing non‐integrin (DC‐SIGN), selectins, as well as toll‐like receptors, leading to signal transductions and downstream pathway activation of various types of immune cells.^[^ ^8^, ^9^, ^10^, ^11^, ^12^ ^]^

The intriguing biological properties of carbohydrates have drawn increasing attentions in applying these molecules in biomedical research and therapeutic developments.^[^ ^13^, ^14^, ^15^, ^16^, ^17^ ^]^ However, the complexity and heterogeneity of natural carbohydrates bring huge difficulties in their production and characterization, thus further structural modification, mechanistic study, and medicinal applications have been significantly limited. To fully exploit the value of carbohydrates and circumvent the disadvantages of materials from natural sources, many efforts have been attempted to prepare homogeneous oligosaccharides and polysaccharides.^[^ ^18^, ^19^, ^20^ ^]^ Moreover, structurally well‐defined synthetic skeletons have been developed to mimic the natural structures.^[^ ^21^, ^22^, ^23^ ^]^ These multivalent systems provide new approaches to design ligands for receptor binding and pathway initiating, but with certain limitations. For example, the broadly applied vesicles/liposomes rely on fatty acids with long hydrocarbon chains that are difficult to be modified.^[^ ^24^ ^]^ The introduction of unnatural components as the cores, such as dendrimers,^[^ ^25^ ^]^ gold‐nanoparticles,^[^ ^26^ ^]^ and other polymeric structures,^[^ ^27^, ^28^ ^]^ may result in undesired receptor binding to suppress the effects from the carbohydrate components, and the potential safety concerns also bring challenges to their further clinical applications.^[^ ^29^, ^30^ ^]^ Therefore, a platform that was derived from natural or biocompatible structural motif, which could be easily constructed and modified, would be of great value.

**Figure 1:**
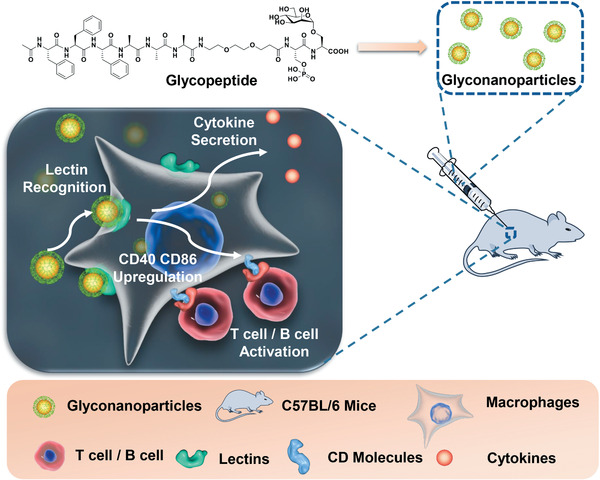
Formation of nanoparticles (NPs) from a self‐assembling glycopeptide conjugate, and immune activation induced by mannosylated NPs in vitro and in vivo.

Considering peptides possess advantages such as easy accessibility, high chemical diversity, and excellent biocompatibility, we envisaged that a self‐assembling peptide system may provide a versatile platform for presenting saccharides in a clustered manner for multivalent interactions to the receptors of interests.^[^ ^31^, ^32^, ^33^, ^34^, ^35^ ^]^ Herein, we report the development of readily accessible self‐assembling glycopeptide sequences with controllable formation of nano structures mimicking the complex polysaccharides, and the evaluation of their biological effects in a series of in vitro and in vivo immunological experiments (**Figure** **1**).

## 2 Results and Discussion

### 2.1 General Design and Synthetic Route

**Figure 2:**
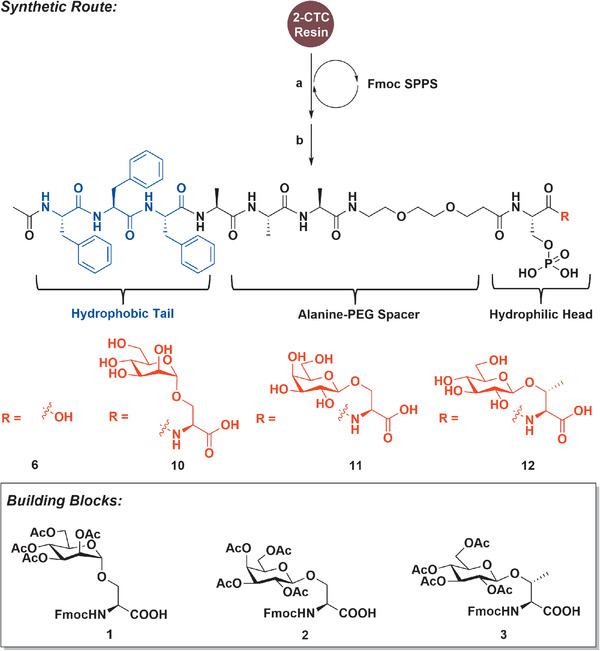
Design and synthesis of self‐assembling sequences: a) Fmoc SPPS: 1) Deprotection: piperidine/DBU/DMF; 2) Coupling: Fmoc‐AA‐OH, glycoamino acids (1‐3) or Fmoc‐Ser(HPO_3_Bzl)‐OH or 9‐[(9H‐fluoren‐9‐ylmethoxy) carbonyl amino]‐4,7‐dioxanonanoic acid, HATU, DIEA. b) Deprotection: 1) Deacetylation: 5% hydrazine hydrate in DMF; 2) Cleavage: TFA/H_2_O/TIPS.

Since structures capable of self‐assembling are usually amphiphilic,^[^ ^36^ ^]^ containing a hydrophobic tail, a hydrophilic head with or without a spacer in between, we selected oligo‐phenylalanines as the core of assembly^[^ ^37^ ^]^ and an alanine‐PEG linker to connect the carbohydrate‐containing hydrophilic head (**Figure** **2**). Moreover, the eukaryotic O‐glycosylation linkages on serine or threonine were chosen to construct the carbohydrate components. Such design afforded proper flexibilities for the possible addition of other natural amino acids with various side chain functionalities modifying the structures and properties, whilst the unnatural components were minimized to only the PEG linker that is widely applied in biotherapeutics.^[^ ^38^ ^]^

Synthetically, the amphipathic peptides could be conveniently prepared utilizing the Fmoc‐based solid phase peptide synthesis (SPPS),^[^ ^39^, ^40^ ^]^ where the O‐glycosylated Ser or Thr cassettes 1–3 could be installed as a building block in each sequence.^[^ ^41^, ^42^ ^]^ Preparative reverse‐phase high performance liquid chromatography may provide the pure product in high quality.

### 2.2 Sequence Screen and Optimization

**Scheme 1:**
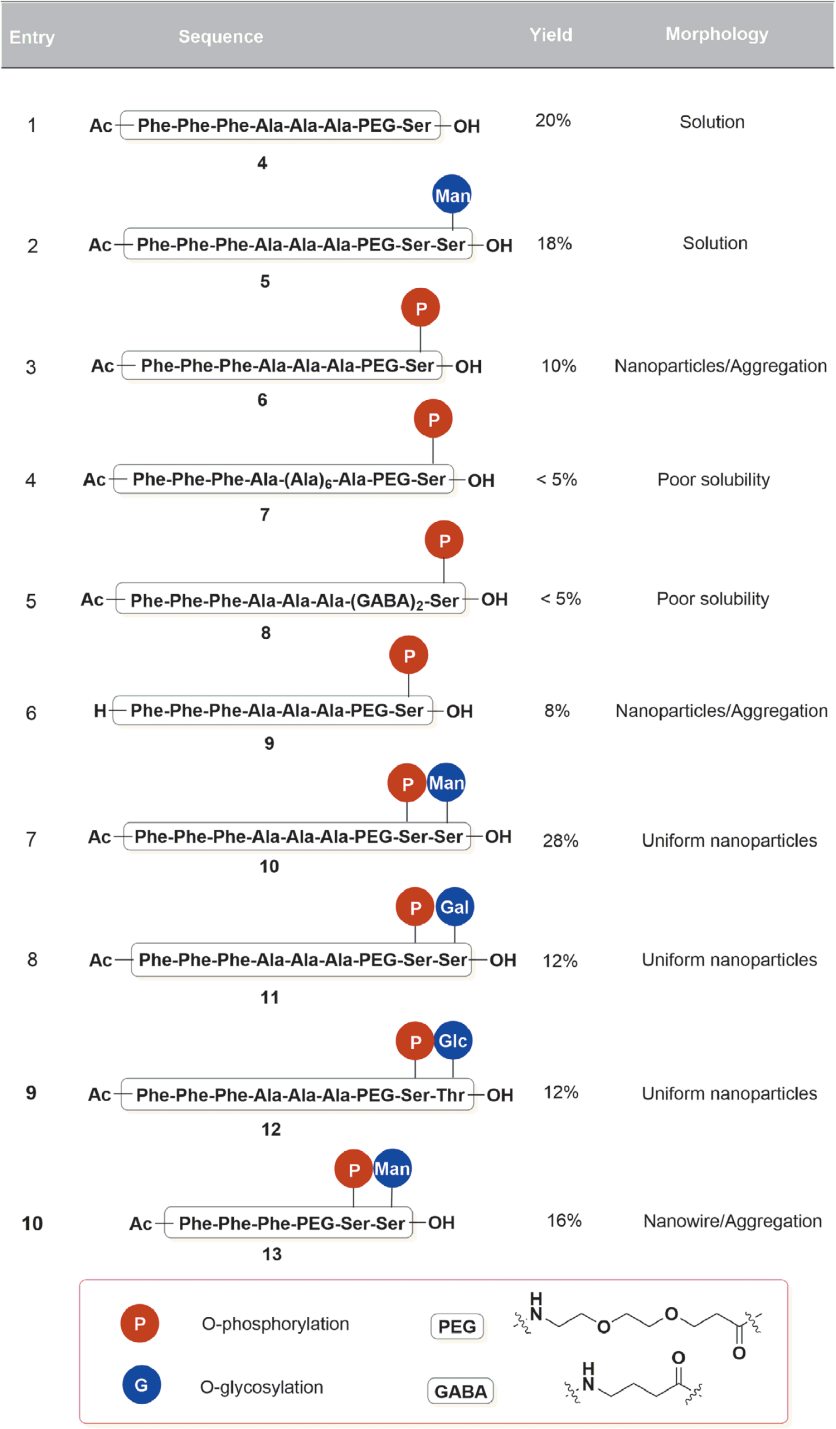
Screen of amphipathic peptide sequences.

With the established synthetic protocol in hand, we first evaluated a sequence containing three repetitive phenylalanines as the hydrophobic end, considering the diphenylalanine moieties are more likely to drive the formation of nanofibers, nanowires, and nanotubes rather than the nanoparticles (NPs).^[^ ^43^, ^44^ ^]^ However, it was noticed that the sequences containing a C‐terminal serine as the hydrophilic end, either with or without an O‐mannose (Man), displayed good solubility and could not form self‐assembled nanostructures (**Scheme** **1**, entry 1 and entry 2). In light of the wide distribution and stabilizing roles of phosphates that present in many biomolecules such as phospholipids and DNA, as well as the use of this biocompatible functionality in synthetic amphiphiles,^[^ ^45^, ^46^, ^47^ ^]^ a phosphoryl serine was incorporated to the sequence (entry 3). The resulted phosphorylated peptide conjugate 6 displayed self‐assembling abilities in PBS buffer, although the formed NPs were not stable enough and significant aggregation was observed. Interestingly, by simply introducing the glycosylation to the phosphorylated sequence led to compound 10 that self‐assembled to uniform nanoparticles (entry 7). In contrast, modifying the sequence by either adding more alanines to extend the sequence (entry 4), or replacing the flexible hydrophilic PEG to a more rigid and hydrophobic GABA linker (entry 5), resulted insoluble peptide conjugates. Notably, capping the N‐terminus with acetyl group was crucial, as the uncapped sequence 9 showed poor self‐assembling ability (entry 6), presumably due to the positively charged amine group under neutral conditions that may sabotage the formation of an amphiphilic system. The optimized sequence was found to robustly generate stable spherical NPs when changing the appended monosaccharide from mannose to galactose (entry 8) or glucose (entry 9). It is worth mentioning that such solid phase‐based synthetic strategy provides great convenience in producing a series of candidate sequences for the required evaluation. Moreover, simply reorganizing the amino acid residues may result in sequences that form nanostructures with different properties and micromorphology. For instance, the glycopeptide sequence omitted the alanines that generate nanowires instead of nanoparticles (entry 10). These results suggest that the amphipathic glycopeptide conjugate may be a general platform that would be of important use for further applications in biomaterials and biomedicines.

### 2.3 Characterization of Nanostructures

**Figure 3:**
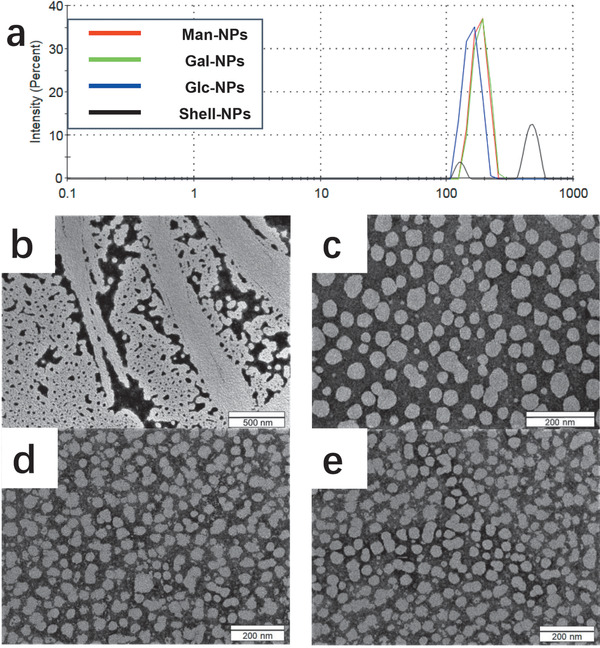
Characterization of self‐assembly nanoparticles: a) Size distribution from dynamic light scattering (DLS) measurement (blue line: glucosylated nanoparticles; green line: galactosylated nanoparticles; red line: mannosylated nanoparticles; black line: shell nanoparticles). b) TEM image of the shell nanoparticles, aggregation could be observed; c) TEM image of mannosylated nanoparticles; d) TEM image of galactosylated nanoparticles; e) TEM image of glucosylated nanoparticles.

With the amphipathic peptides in hand, we evaluated their self‐assembly using zeta potential measurements, transmission electron microscopy (TEM), and dynamic light scattering (DLS), to explore the biophysical and structural properties of the formed nanostructures. Experimental data based on intensity DLS indicate that the peptide conjugates self‐assembled in aqueous solution and formed NPs, which are more evenly distributed than the ones generated from the non‐glycosylated sequences (**Figure** **3**a). Moreover, the generated glycosylated nanoparticles displayed higher zeta potential values (Figure S1, Supporting Information), suggesting better stabilities than that of the particles without glycosylation.

TEM images of glycosylated nanoparticles further demonstrated their uniform morphology (Figure 3c–e), whilst the aggregates derived from the non‐glycosylated peptide conjugates were clearly observed under the microscopy (Figure 3b). The nanowire formation from dispersing peptide 13 in aqueous buffer could also be observed under TEM as well (Figure S16, Supporting Information). It is noteworthy that the particle size estimated by microscopy is smaller than the data obtained based on DLS experiments. This is because the introduction of phosphate groups and carbohydrates, as well as the flexible hydrophilic PEG linker, may promote the formation of a thick hydration layer for each NP,^[^ ^48^, ^49^, ^50^ ^]^ which would be dehydrated to cause a shrinkage of size during the sample preparation for TEM. Considering the DLS measurements were performed in aqueous buffer, the results thus obtained for the glycosylated nanoparticles (≈100–200 nm) should be closer to the actual size of these NPs under physiological environments.^[^ ^51^ ^]^ Moreover, such size distribution are suitable for lymph nodes delivery and could be a crucial factor for immune activation.^[^ ^52^ ^]^ Along this line, we further investigated the biological activities of the obtained glycosylated nanoparticles to exemplify their potential biomedical applications.

### 2.4 In Vitro Macrophage Stimulation by Glycopeptides‐Assembled Nanoparticles

**Figure 4:**
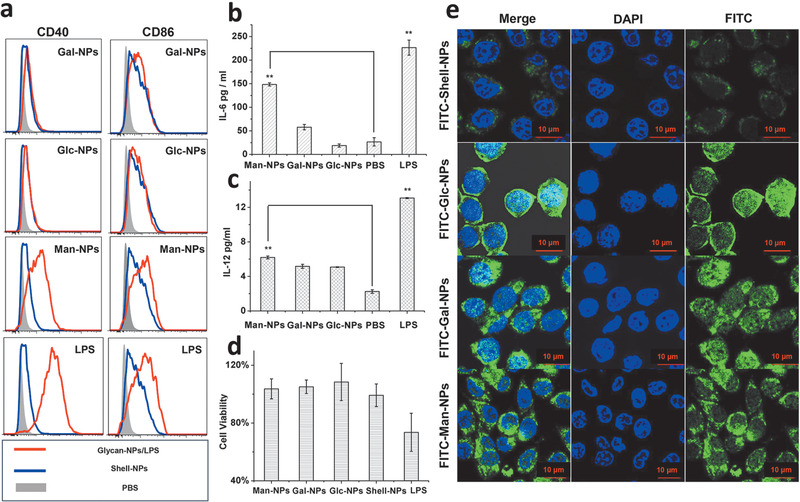
Activation of RAW 264.7 macrophages by nanoparticles [galactosylated nanoparticles (Gal‐NPs), glucosylated nanoparticles (Glc‐NPs), mannosylated nanoparticles (Man‐NPs), non‐glycosylated shell nanoparticles (Shell‐NPs), and lipopolysaccharide (LPS, as positive control)]: RAW 264.7 macrophages are stimulated by different glycopeptides for 48 h, the activation of macrophage is characterized by a) CD40 and CD86 expression levels using flow cytometry, b) IL‐6, and c) IL‐12 in cell culture supernatant using ELISA. d) CCK‐8 test of the cell viability after the incubation with NPs or LPS for 72 h. e) Confocal microscopy image of RAW 264.7 macrophages incubated for 48 h with fluorescently labeled none‐glycosylated NPs (first row), glucosylated NPs (second row), galactosylated NPs (third row), and mannosylated NPs (fourth row). Cell nucleus were stained by DAPI (blue) and nanoparticles were labeled by FITC (green). Data are reported as mean ±SD, **p < 0.01.

Bearing in mind the increasing research interests and medical applications regarding immunotherapy,^[^ ^53^, ^54^, ^55^, ^56^ ^]^ as well as the immunomodulating abilities of a number of natural carbohydrates and their conjugates reported in recent years,^[^ ^57^, ^58^, ^59^ ^]^ we wondered whether the developed glycopeptides self‐assembly may mimic the natural polysaccharides and be utilized in adjuvating immunotherapy. Thus, a RAW 264.7 macrophage cell line was utilized to evaluate the activation of immune cells by tailored nanoparticles, wherein upregulated co‐stimulatory receptors would be expected upon positive stimulation.^[^ ^60^, ^61^ ^]^ Flow cytometry analysis indicated that mannosylated nanoparticles significantly increased the expression levels of both CD40 and CD86 on macrophage while the groups of glucosylated, galactosylated, and non‐glycosylated nanoparticles displayed insignificant activation effects (**Figure** **4**a). Furthermore, significantly increased expression levels of two pro‐inflammation cytokines, interleukin‐6 (IL‐6) and interleukin‐12 (IL‐12) were induced by the mannosylated nanoparticles (Figure 4b,c), as quantified by the enzyme‐linked immunosorbent assay (ELISA),^[^ ^62^, ^63^ ^]^ suggesting that the mannosylated nanoparticles could effectively activate the macrophage. In consideration of a previous report from the Becer group showing that star‐shaped mannose glycopolymers can effectively activate dendritic cells and lead to significant change of the secretion levels of interleukin‐10 and interleukin‐12p70,^[^ ^64^ ^]^ together these results further highlight the pivotal roles of mannose receptors in the stimulation of antigen presenting cells. Notably, although the positive control liposaccharide (LPS) in our experiments shows higher stimulation activity than that of the mannosylated NPs, severe cytotoxicity was observed in the LPS‐treated group (Figure 4d). In contract, the glycosylated NPs displayed no obvious cytotoxicity, suggesting much safer application while being used as a component of drugs or vaccines.

In order to visualize the possible interactions between nanoparticles and immune cells, the glycopeptide conjugates were labeled using covalently linked fluorescein isothiocyanate (FITC) at the N‐terminus, and the resulted fluorescent NPs were incubated with the RAW 264.7 macrophages for 72 h and then analyzed using the confocal microscopy. Comparing to the non‐glycosylated NPs, the glyconanoparticles displayed obvious accumulation on the surface of cells (Figure 4e). Particularly in the group with mannosylated materials, significant number of labeled nanoparticles were observed inside the cells. These results suggest that the nanoparticles may be recognized by the receptors on the surface of macrophages and could enter cell plasma (presumably via the endocytosis pathway), which lead to the immune activation.

**Figure 5:**
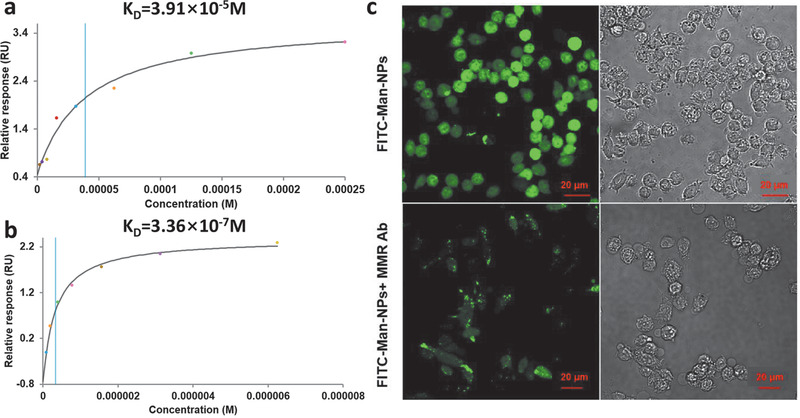
Mechanism study of macrophage activation: SPR analysis of a) unmodified mannose binds to ConA or b) Man‐NPs bind to ConA. c) Confocal microscopy images of RAW 264.7 macrophages treated with FITC‐Man‐NPs without MMR Ab (top) or with MMR Ab (bottom), respectively. Nanoparticles were green under fluorescence (left). The macrophage cells shown in grey scale at the bright field (right).

To get a mechanistic insight of the immune activation in macrophage induced by mannosylated nanoparticles, a series of experiments were conducted to verify whether the self‐assembled glyconanoparticles may have interacted with the PRRs, similar to those natural polysaccharides such as mannans. We first evaluated the binding affinities of nanoparticles toward concanavalin A (ConA), a lectin that can recognize the terminal *α*‐D‐mannoses and *α*‐D‐glucoses of complex carbohydrates, and has been utilized in a number of binding assays of membrane C‐type lectin.^[^ ^65^, ^66^ ^]^ Surface plasmon resonance (SPR) experiments indicate that the nanoparticles generated from mannosylated peptide conjugate 7 show approximately two orders of magnitude higher affinity to the immobilized ConA than the unmodified mannose alone (**Figures** **5**a,b). The SPR curves of Man‐NPs exhibit fast association and dissociation rates (Figure S17, Supporting Information),^[^ ^67^, ^68^ ^]^ and the concentration dependency and saturation of receptors could be observed, which rules out the possibility of non‐specific bindings.^[^ ^67^ ^]^ Notably, examples of mannose‐containing glycopolymers have shown extremely high affinity toward lectins such as DC‐SIGN,^[^ ^69^ ^]^ indicating that the binding value in SPR may be affected by structure and size of both the ligand and receptor.^[^ ^68^ ^]^ These literature precedents suggest that further optimization of the self‐assembling glycopeptide conjugates, such as adjusting the length of PEG linker and the number of incorporated mannoses, may be beneficial for an enhanced binding affinity in the modulation of specific target receptors. At this stage, the obtained results demonstrate that the assembled nanoparticles from the peptide conjugates show much stronger binding to ConA in comparison to the unmodified mannose, presumably through multivalent interactions, which also underscores a potential cluster effect of the Man‐NPs similar that of those related polysaccharides.

Aiming to further identify the possible receptors that the Man‐NPs bind to, RAW 264.7 cells were treated with the FITC‐Man‐NPs with or without the addition of macrophage mannose receptor antibody (MMR Ab), respectively, and incubated for 24h. Examination using confocal microscopy showed that MMR Ab could evidently interfere the binding of Man‐NPs to macrophages (Figure 5c). This result suggests that the macrophage mannose receptor (MMR) may be one of the major targets that the Man‐NPs bind to, and interaction between the MMRs and Man‐NPs may simulate the downstream activation of macrophage cells.

### 2.5 In Vivo Evaluation of the Glycosylated Nanoparticles

**Figure 6:**
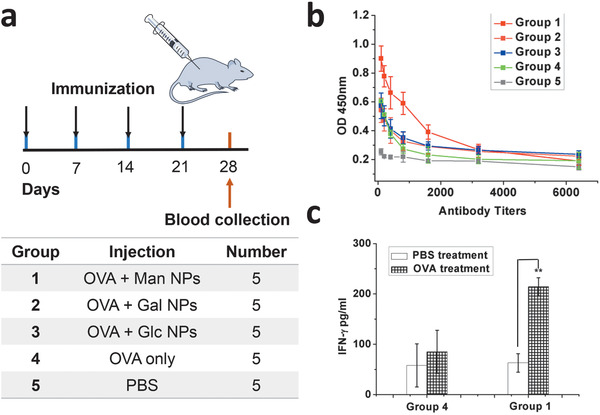
Immunological evaluation with OVA model antigen: a) Groups of vaccination. b) IgG antibody titers of different groups. c) IFN‐*γ* production in the cell media of splenocytes from immunized mice after in vitro re‐stimulation. Data are reported as mean ± SD, **p < 0.01.

In order to demonstrate the immunostimulatory capabilities in vivo, the glyconanoparticles were evaluated as an adjuvant in vaccination of mice. Ovalbumin (OVA) was chosen as a model antigen, which has been broadly applied in the evaluation of vaccine adjuvants. Groups of five C57BL/6 mice were vaccinated subcutaneously, and each group was given the OVA protein mixed with glycosylated nano particles, or OVA only, or PBS buffer as the control group, respectively (**Figure** **6**). After four immunizations at weekly intervals (days 0, 7, 14, 21), mouse sera were collected on day 28 and analyzed for the production of anti‐OVA IgG antibodies. As assessed by ELISA, the group of mice vaccinated with the combination of mannosylated nanoparticles and OVA antigen elicited the highest IgG antibodies titers (Figure 6b). In contrast, antibody levels of the groups that vaccinated with galactosylated or glucosylated NPs as stimulators did not show significant difference comparing to the group that vaccinated with OVA only, suggesting that the mannose modification is necessary for such nanostructures to induce immunostimulatory response in vivo. These results are also in accord to the in vitro observations.

To further investigate the activation ability of mannosylated nanoparticles, the spleen cells from mice that vaccinated for 4 weeks with mannosylated NPs plus OVA (group 1) and OVA only (group 4) were stimulated by OVA for 48 h in vitro. The expression levels of IFN‐*γ* in cell media with or without OVA stimulation was measured by ELISA (Figure 6c). Upregulation of IFN‐*γ* in both groups indicates an OVA antigen‐specific memory response, and the group with mannosylated NPs displayed enhanced level of IFN‐*γ* expression (over threefold higher than the control group), further confirming the immunostimulating capability of the mannosylated nanoparticles.

## 3 Conclusions

Self‐assembling peptides have been applied in a broad range of drug delivery, antibacterial material, and tissue engineering, showing their advantageous roles in biomedical applications.^[^ ^70^, ^71^, ^72^ ^]^ However, very limited studies have been conducted on carbohydrate associate self‐assembling process. This glycopeptide‐based self‐assembly we have developed provided a readily accessible and maneuverable platform for constructing nanostructures that may be of great importance, such as applications in displaying simple carbohydrates in a multivalent format that excellently mimicked the natural complex polysaccharides. As the recent advances in chemical synthesis have afforded many efficient methods to construct and modify glycopeptides, utilizing such highly biocompatible structures derived from natural monosaccharides and amino acids to construct nanostructures represents an advantageous approach in biomedical research and therapeutic developments.

In this study we have demonstrated that the self‐assembling glycopeptide conjugates could be used in generating a series of multivalently glycosylated nanoparticles, which may be able to interact with lectins that often targeted by complex natural carbohydrates. The immunostimulatory activities of these glyconanoparticles have been evaluated both in vitro and in vivo. The results indicate that the mannose‐modified NPs could act as an immune activator in both macrophage cell culture and mice vaccination, presumably through binding to MMRs as one of the major activation pathways. With the ease of constructing the glycopeptide sequences, it was envisioned that more diverse oligosaccharide epitopes could be introduced, which may result in different immune activation pathways. Such self‐assembling strategy may also allow for further discovery of more well‐designed and precisely modified glyco‐nanostructures.

## 4 Experimental Section

##### Preparation of FITC‐Labeled NPs

The peptide and FITC (equiv ratio = 1:2) were dissolved in pH 9 buffer (7.56 g NaHCO_3_, 1.06 g Na_2_CO_3_, and 7.36 g NaCl per litter), and stirred in dark overnight. Unreacted FITC was removed by ultrafiltration, and the resulted NPs solution was directly used in the followed studies without further purification.

##### Characterization of Nanostructures

ZETASIZER NANO ZSP was employed to perform the DLS experiment. JEM1200EX transmission electron microscope was employed to directly observe the nanostructures.

##### Cell Culture

RAW 264.7 cell line was obtained from Cell Resource Center, IBMS, CAMS/PUMC, and maintained in DMEM media (corning) supplemented with 10% fetal bovine serum (FBS), 100 µg mL^−1^ streptomycin and 100 unit mL^−1^ penicillin.

##### Cell Viability

RAW 264.7 cells were diluted into 1 × 10^5^ cells mL^−1^ and 100 µL media was added to each well (1 × 10^4^ cells) in the 96‐well plate. For measurement, different groups of nanoparticles (Shell‐NPs, Man‐NPs, Gal‐NPs, and Glc‐NPs) and LPS solution were prepared in medium and added in triplicate into a 96‐well plate with a volume of 100 µL per well (final concentration = 100 µg mL^−1^). After incubation for 72 h at 37 °C, the CCK‐8 (Solarbio) method was employed to measure the cell viability. The corresponding OD450 was measured by Multiskan GO (Thermo Scientific) microplate reader.

##### Flow Cytometry Analysis

RAW 264.7 cells were diluted to 1 × 10^5^ cells mL^−1^ and 1 mL media was added to each well (1 × 10^5^ cells) in the 12‐well plate. Different groups of nanoparticles (Shell‐NPs, Man‐NPs, Gal‐NPs, and Glc‐NPs) and LPS solution were prepared in medium and added in triplicate into a 12‐well plate with a volume of 1 mL per well (final concentration = 100 µg mL^−1^). After incubation for 48 h at 37 °C, the media was removed. After washing with DPBS, the cell was incubated with PE labeled antibodies (anti‐CD40 and anti‐CD86) diluted in a 1:100 ratio at 0 °C for 1 h. After washing with DPBS, cells of different groups were collected for the flow cytometry analysis on flow cytometer CytoFLEX.

##### SPR Experiments

The interaction between ConA protein and Man‐NPs as well as unmodified mannose was measured on a Biacore 8k (GE Healthcare) instrument at 25 °C in a running buffer containing PBS, and 0.05% surfactant P20. ConA protein (Solarbio) was immobilized on a sensor chip (CM5) using a typical amine coupling procedure. No regeneration of the chip surface was required between different analyte injections.

##### Binding Experiments of FITC‐NPs under Confocal Microscopy

RAW 264.7 cells were diluted to 1 × 10^5^ cells mL^−1^ and 1 mL media was added to each well (1 × 10^5^ cells) in the 12‐well plate. Different groups of FITC‐labeled nanoparticles (FITC‐Shell‐NPs, FITC‐Man‐NPs, FITC‐Gal‐NPs, and FITC‐Glc‐NPs) were prepared in medium and were added in triplicate into a 12‐well plate. After incubation for 48 h at 37 °C, the media was removed. After washing with DPBS, fixed with 4% paraformaldehyde for 20 min, and treated with Triton x100 for 10 min, the nucleus (DNA) was stained by DAPI for 3 min. The samples were immediately examined and analyzed by confocal microscopy in DAPI and FITC channels.

##### Vaccination of Mice

6‐week‐old C57BL/6 mice were purchased from Peking University Health Science Center. 25 mice were divided into 5 groups, each group was given intravenous injection of OVA protein mixed with glycosylated nanoparticles, or OVA only, or PBS buffer. 20 µg of antigen (OVA protein) was given to a mouse each time. After vaccination for four times at days 0, 7, 14, 21, the mice serum was collected at day 28 for IgG titers. All experiments were conducted at Peking University in accordance with National Institutes of Health guidelines and were approved by the Institutional Animal Care and Use Committee.

##### Antibody (IgG) Titers

High‐binding 96‐Well ELISA plates were coated with OVA protein (20 µg mL^−1^) in a Na_2_CO_3_/NaHCO_3_ buffer (pH = 9.6) at 4 °C overnight. After being washed with 0.05% tween‐PBS solution, the plates were blocked with 3% BSA solution. The antiserum was then diluted to different concentrations using 1% BSA in PBS and added to the plates (100 µL well^−1^) and incubated for 2 h at 37 °C. The plates were washed for three times, and incubated with rabbit anti‐mouse IgG‐Peroxidase antibodies (SBA, 1:2000 dilution) for 1 h at 37 °C. Then the plates were further washed for three times, followed by the addition of TMB substrate (Solarbio), and reacted at room temperature for 20 min in dark. The corresponding OD_450_ was measured by Multiskan GO (Thermo Scientific) microplate reader.

##### ELISA Measurement for IFN‐*γ* in Spleen Cell Culture

After immunization, the spleens of the immunized mice were removed under sterile conditions and suspended in sterile cold PBS. After centrifugation, the cell suspension was diluted to 2 × 10^6^ cells mL^−1^ in RPMI 1640 supplemented with 10% FBS, 100 µg mL^−1^ streptomycin and 100 unit mL^−1^ penicillin, and cultured overnight. After removal of the non‐adherent cells, the medium was changed, and the OVA protein in buffer and PBS buffer only were added to different wells, respectively, and cultured for 48 h. The culture media was then collected and diluted for the ELISA tests. Following the instructions of ELISA kit, the samples, anti‐IFN‐*γ* antibody, and chromogenic substrates were added in order. The corresponding OD_450_ was measured by Multiskan GO (Thermo Scientific) microplate reader.

##### Statistical Analysis

Data are reported as mean ± SD and the statistical significance is determined using one‐way ANOVA analysis. *p < 0.05, **p < 0.01.

## Conflict of Interest

The authors declare no conflict of interest.

## Supporting information

Supporting Information
