# Supplementary material for: Self‐Assembling Glycopeptide Conjugate as a Versatile Platform for Mimicking Complex Polysaccharides
Source: Adv Sci (Weinh). 2020 Jul 2;7(16):2001264. doi: 10.1002/advs.202001264 (PMC7435236; doi:10.1002/advs.202001264)
Supplement: Supplementary file 1 — Supporting Information [file ADVS-7-2001264-s001.pdf]

Supporting Information

Self-Assembling Glycopeptide Conjugate as a Versatile Platform for Mimicking Complex Polysaccharides

Hanxuan Wang[1,3], Zhichao Liu[1,3], Chuanjing An[1], Haoting Li[1], Fanlei Hu[2] & Suwei Dong[1*]

[1]State Key Laboratory of Natural and Biomimetic Drugs, and Department of Chemical Biology, School of Pharmaceutical Sciences, Peking University, Xueyuan Road No. 38, Haidian District, Beijing 100191, China.

[2]Department of Rheumatology and Immunology, Peking University People's Hospital & Beijing Key Laboratory for Rheumatism Mechanism and Immune Diagnosis (BZ0135), Beijing, China.

[3]These authors contributed equally: Hanxuan Wang, Zhichao Liu.

E-mail: dongsw@pku.edu.cn

Table of Contents

Abbreviations S3

I. General Information S5

II. Procedures for the Preparation of Monosaccharides, Amino Acids and Derivatives S7

III. General Procedures for Peptide Synthesis S11

IV. Preparation and Characterization of Peptide Sequence S14

V. Preparation of FITC-Labeled Nanoparticles S23

VI. Characterization of Nano Particulate S29

VII. *In vitro* Study of Glycosylated Nanoparticles S31

Abbreviations

**Ac**	Acetyl
**Ala**	Alanine
**BSA**	Bovine serum albumin
**CCK-8**	Cell Counting Kit-8
**CD**	Cluster of differentiation
**DAPI**	4',6-diamidino-2-phenylindole
**DBU**	1,8-Diazabicyclo[5.4.0]undec-7-ene
**DCM**	Dichloromethane
**DIEA**	Ethyldiisopropylamine
**DLS**	Dynamic light scattering
**DMF**	N, N’-Dimethylformamide
**DPBS**	Dulbecco's phosphate buffered saline
**ELISA**	Enzyme linked immunosorbent assay
**ESI-MS**	Electrospray ionization mass spectrometry
**FACS**	Fluorescence activated cell sorting
**FITC**	Fluorescein isothiocyanate
**Fmoc**	Fluorenylmethyloxycarbonyl
**Gal**	Galactose
**Glc**	Glucose
**HATU**	1-[Bis(dimethylamino)methyl-ene]-1H-1,2,3-triazolo[4,5-b] pyridinium 3-oxid hexafluorophosphate
**HPLC**	High performance liquid chromatography
**LC-MS**	Liquid chromatography-mass spectrometry
**IFN**	Interferon
**IL**	Interleukin
**LPS**	Lipopolysaccharide
**Man**	Mannose
**MMR**	Macrophage mannose receptor

**NMR**	Nuclear magnetic resonance
**OVA**	Ovalbumin
**Oxyma**	Ethyl (hydroxyimino)cyanoacetate
**PBS**	Phosphate buffer saline
**PE**	Phycoerythrin
**Phe**	Phenylalanine
**rt**	Room temperature
**Ser**	Serine
**SPPS**	Solid phase peptide synthesis
**SPR**	Surface plasmon resonance
***t*-Bu**	tert-Butyl
**TEA**	Triethylamine
**TEM**	Transmission electron microscope
**TFA**	Trifluoroacetic acid
**Thr**	Threonine

I. General Information

1.1 Materials and Methods

HPLC grade methylene chloride, diethyl ether, toluene, and DMF were purchased from Fisher, Acros, J&K, and Oceanpak, and were purified and dried by passing through a PURE SOLV® solvent purification system (Innovative Technology, Inc.) when anhydrous solvents were required. Other reagent grade solvents for chromatography were purchased from Beijing Tongguang Fine Chemicals Company. Molecular sieves (4 Å, power) were pre-activated in an oven at 65 ℃ overnight and further flame-dried before being used in the reactions. All other reagents and relevant catalysts were purchased from Sigma-Aldrich, NovaBiochem, GL Biochem, Acros, TCI, Adamas, Innochem, J&K, Alfa, and Energy, and were used without further purification. Ultra-pure argon (≥99.999%) was used when inert reaction conditions were required. All antibodies were purchased from biolegend and Proteintech. All ELISA kit were purchased from JSKT and JIMEI.

Analytical thin layer chromatography was performed using 0.25 mm silica gel 60-F plates (Merck). Flash chromatography was performed using 200-300 mesh silica gel (Qingdao Haiyang Chemical Co., Ltd.). Yields refer to chromatographically and spectroscopically pure materials unless otherwise stated. ^{1}H NMR spectra were recorded at 400 MHz at ambient temperature with $CDCl_3$ (Cambridge Isotope Laboratories, Inc.) as the solvent unless otherwise stated. ^{13}C NMR spectra were recorded at 100 MHz at ambient temperature with $CDCl_3$ as the solvent unless otherwise stated. High-resolution mass spectra were obtained in the Chemical Instrumentation Center, Peking University Health Science Center using a Waters Q-TOF mass spectrometer (Xevo G2 Q-TOF). Zeta potential and diameter were measured on ZETASIZER NANO ZSP. TEM pictures were obtained using JEM1200EX transmission electron microscope. The FACS experiments were performed using CytoFLEX. The absorbance of cell viability assay and ELISA were detected on a Multiskan™ GO (Thermo Scientific™) microplate reader. All the confocal images were recorded using Nikon A1R / A1 confocal laser microscope system. C57BL/6 mice were purchased from purchased from Peking University Health Science Center and mice were well cared for and approved by Peking University Health Science Center

1.2 HPLC

All HPLC separations involved a mobile phase of 0.05% (*v/v*) TFA in water (solvent A) and 0.04% (*v/v*) TFA in MeCN (solvent B) unless otherwise stated. All peptide UV traces were recorded under the wavelength of 210 nm and 220 nm in analytical HPLC-MS, analytical HPLC and preparative HPLC.

Analytical HPLC-MS chromatographic separations were performed using a Waters Alliance e2695 Separations Module, an SQ Detector, and a Waters 2489 UV/Visible (UV/Vis) Detector equipped with an Agilent C18 column (5.0 μm, 4.6 × 150 mm) at a flow rate of 0.4 mL/min. The wavelengths of UV-detector were set to 210 nm and 220 nm.

Analytical HPLC chromatographic separations were performed using an Agilent Technologies 1260 Infinity LC system equipped with an Agilent C18 column (5.0 μm, 4.6 × 150 mm) at a flow rate of 0.4 mL/min. The wavelengths of UV-detector were set to 210 nm and 220 nm.

Preparative HPLC separations were performed using a Hanbon Sci. & Tech. NP7005C solvent delivery system and a Hanbon Sci. & Tech. NU3010C UV detector equipped with an Agilent Eclipse XDB-C18 column (7.0 μm, 21.2 × 250 mm) at a flow rate of 16 mL/min. The wavelengths of UV-detector were set to 210 nm and 220 nm.

II. Procedures for the Preparation of Monosaccharides, Amino Acids and Derivatives

2.1 Preparation of Mannose Derivatives

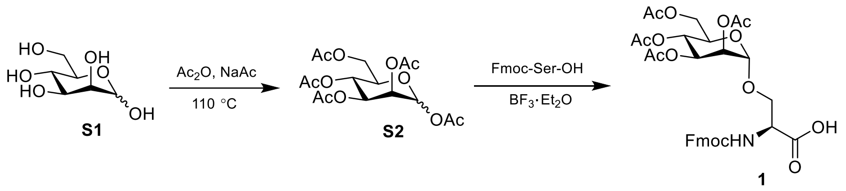

Scheme S1. Synthesis of O-mannosyl serine derivatives **1**. [S1]

Mannose pentaacetate (S2): The mixture of mannose **S1** (2 g, 11.10 mmol) and NaOAc (1.2 g, 14.63 mmol) in Ac_2O (20 ml) was stirred at 110 °C for 2 h. The solution was concentrated to remove Ac_2O, poured into ice-cold aq. $NaHCO_3$, extracted with DCM, washed with brine, and dried with Na_2SO_4. The resulting solution was filtered and concentrated to give 5.8 g colorless syrup **S2**.

Fmoc-Ser (Ac$_4$Manα)-OH (1): Fmoc-Ser-OH **S3** (1 g, 3 mmol) and mannose pentaacetate **(S2)** (1.8 g, 2.5 mmol) were dissolved in dry DCM (10 ml) under argon. The solution was cooled to 0 °C, and $BF_3 \cdot OEt_2$ (0.94 ml, 7.5 mmol) was added dropwise. The reaction was stirred overnight in room temperature. Then the reaction was monitored by TLC. The reaction was diluted with ethyl acetate and washed with water. The organic layer was dried over $MgSO_4$, filtered, and concentrated. The residue was purified by flash chromatography on a silica gel column; elution with a gradient of ethyl acetate in hexanes (25%-50%) with 1% acetic acid to afforded compound **1** (921 mg, 56%) as white powder. ^1H and ^{13}C NMR for compound **1** were identical to data reported in literature. [S1]

[S1] L. Chen, Z. Tan, *Tetrahedron Lett.* **2013**, *54*, 2190-2193.

2.2 Preparation of Galactose Derivatives

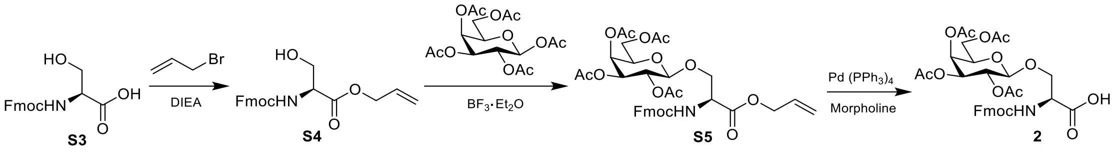

Scheme S2. Synthesis of O-galactosyl serine derivatives **2**. [S2]

Fmoc-Ser-OAll (S4)*:* Fmoc-Ser-OH **S3** (1 g, 3.06 mmol) was dissolved in DMF (15 ml), and N, N-Diisopropylethylamine (1 ml, 6.12 mmol) and allyl bromide (0.32 ml, 3.67 mmol) was added dropwise with ice bath. The reaction was stirred overnight and then monitored by TLC. The mixture was diluted with ethyl acetate, then washed with water three times, and washed with brine once. The organic layer was dried over $MgSO_4$, filtered, and concentrated. The residue was purified by flash chromatography on a silica gel column; elution with a gradient of ethyl acetate in hexanes (25%) afforded **S4** (1 g, 89%) as colorless oil.

Fmoc-Ser (Ac$_4$Galβ) Allyl Ester (S5)*:* Fmoc-Ser-OAll **S4** (600 mg, 1.63 mmol) and β-D-galactose pentaacetate (1.4 g, 1.96 mmol) were dissolved in dry DCM (8 ml) under argon. The solution was cooled to 0°C, and $BF_3{\cdot}OEt_2$ (0.62 ml, 4.89 mmol) was added dropwise. The reaction was stirred overnight. Then the reaction was monitored by TLC. The reaction was diluted with ethyl acetate, washed with water. The organic layer was dried over $MgSO_4$, filtered, and concentrated. The residue was purified by flash chromatography on a silica gel column; elution with a gradient of ethyl acetate in hexanes (25%-50%) afforded **S5** (864 mg, 76%) as white foam.

Fmoc-Ser (Ac$_4$Galβ)-OH (2)*:* Fmoc-Ser (Ac$_4$Galβ) Allyl Ester **S5** (864 mg, 1.24 mmol) and Pd $(PPh_3)_4$ (72 mg, 0.062 mmol) were dissolved in dry THF (6 ml) under argon. Morpholine (0.2 ml, 2.36 mmol) was added dropwise. The reaction was stirred for 20 min. The reaction was monitored by TLC. After concentrated, the residue was purified by flash chromatography on a silica gel column; elution with a gradient of ethyl acetate in hexanes (25%-50%) with 1% acetic acid afforded **2** (726 mg, 89%) as white powder. ^{1}H and ^{13}C NMR for compound **2** were identical to data reported in literature.[S2]

[S2] H. Lin, D. A. Thayer, C.-H. Wong, C. T. Walsh, *Chem. Biol.* **2004**, *11*, 1635-1642.

2.3 Preparation of Glucose Derivatives

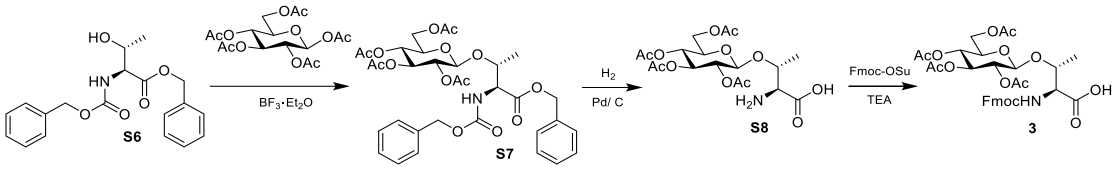

Scheme S3. Synthesis of O-glucosyl threonine derivatives **3**. [S3]

Z-Thr (Ac4Glcβ)-OBzl (S7): β-D-glucose pentaacetate (5 g, 7.0 mmol) and Z-Thr-OBzl **S6** (1.23 g, 3.58 mmol) were dissolved in anhydrous DCM (4.25 ml). The solution was cooled to 0 °C and ice-cold boron trifluoride ethyl etherate (4.5 ml, 35.8 mmol) was added dropwise. Moisture should be avoided during the reaction. The mixture was stirred at room temperature for 24 h, by monitoring the reaction by TLC, and poured into ice water. The aqueous phase was separated, extracted with dichloromethane and the combined extracts were washed with ice-cold 2.5% sodium hydrogen carbonate and water, dried and evaporated to dryness. The oily residue, still containing the excess of sugar, was triturated with light petroleum, dried in vacuo and directly submitted to catalytic hydrogenolysis as described below.

Thr (Ac4Glcβ)-OH (S8): Crude Z-Thr (Ac4Glcβ)-OBzl **S7** was dissolved in glacial acetic acid (20 ml) and hydrogenated for 20 h. The catalyst was removed by filtration and the filtrate diluted with water and extracted with ethyl acetate. The aqueous phase was evaporated to dryness in vacuo at 30 °C and the residue triturated several times with ether and dried. The residue was purified by HPLC and afforded the **S8** (498 mg, 31% in two steps).

Fmoc-Thr (Ac4Glcβ)-OH (3): Thr (Ac4Glcβ)-OH **S8** (498 mg, 1.11 mmol) and Fmoc N-hydroxy succinimide ester (412 mg, 1.22 mmol) was dissolved in dry DCM (7 ml). The solution was cooled to 0 °C, and triethylamine (0.31 ml, 2.22 mmol) was added dropwise. The reaction was stirred overnight in room temperature. Then the reaction was monitored by TLC. The reaction was diluted with ethyl ester, washed with water. The organic phase was dried over $MgSO_4$, filtered, and concentrated. The residue

---

[S3] F. Filira, L. Biondi, F. Cavaggion, B. Scolaro, R. Rocchi, *Int. J. Pept. Protein Res.* **1990**, *36*, 86-96.

was purified by flash chromatography on a silica gel column; elution with a gradient of ethyl acetate in hexanes (25%-50%) with 1% acetic acid afforded the **3** (559 mg, 75%) as white powder. ^{1}H and ^{13}C NMR for compound **3** were identical to data reported in literature. [S3]

III. General Procedures for Peptide Synthesis

3.1 Preparation of amino acid pre-loaded resin and determination of resin loading[S4]

3.1.1 Pre-load an amino acid to 2-chlorotritylchloride resin

The C-terminal amino acid residue was loaded to 2-chlorotritylchloride resin before Fmoc-SPPS following the general procedure below.

To a mixture of Fmoc-amino acid (1.2 equiv) and 2-chlorotritylchloride resin was added dry DCM (approx. 10 mL per gram of resin) and DIEA (2.0 equiv). The reaction was agitated for overnight. The resin was collected and washed with 17/2/1 (*v*/*v*/*v*) of DCM/MeOH/DIEA (× 3), DCM (× 3), DMF (× 2), DCM (× 3), and dried *in vacuo* for 12 hours before the loading test.

3.1.2 Determination of resin loading [S5]

Dry Fmoc amino-acid resin (W_{resin} = approx. 5 μmol with respect to Fmoc) was weighted into a clean test tube, followed by the addition of 2 mL of 2% DBU in DMF. The mixture was agitated gently for 30 min, and then diluted to 10 mL with CH_3CN. 2 mL of the resulting solution was taken out and diluted to 25 mL in a 50 mL centrifuge tube as the test solution. A reference solution was prepared in the same manner without the addition of resin.

The silica UV cell was filled with reference solution to blank the U.V. spectrophotometer. The solution in the silica UV cell was changed to the test solution after washing with the test solution for three times. The optical density at 304 nm was recorded for three times and the average value was calculated as Abs_{sample}. The Fmoc loading of resin could be calculated using the equation below:

$$\text{Fmoc loading: mmol/g} = Abs_{sample} * 125/(\varepsilon_{304\,nm} * W_{resin})$$

The extinction coefficient of dibenzofulvene at 304 nm is $\varepsilon_{304\,nm} = 7624\ M^{-1}cm^{-1}$.

[S4] Peptide Synthesis, 2010/2011 Catalog, Merck.

[S5] Gude, M.; Ryf, J.; White, P. D. *Lett. Pept. Sci.* **2002**, *9*, 203-206.

3.2 Solid-Phase Peptide Synthesis

3.2.1 Manual Solid-Phase Peptide Synthesis

Deprotection: Peptide resin was treated with piperidine/DMF (20 : 80, *v/v*) for 2 times, and washed with DMF (× 3), DCM (× 3) and DMF (× 3).

Amino acid coupling: Fmoc-L-Ala-OH or Fmoc-L-Phe-OH (4 equiv), HATU (4 equiv) was dissolved in 4 mL of DMF, to which DIEA (4 equiv) was added. The amino acid was preactivated for 1 min, then the solution was added to the resin. After agitated for 20 min, the resin was washed with DMF (× 3), DCM (× 3) and DMF (× 3), and same coupling cycle was repeated. For amino acids after the first Phe, the coupling cycle was repeated as needed.

Commercial available Fmoc-Ser(PO(OBzl)OH)-OH (2 equiv), HATU (2 equiv) was dissolved in 4 mL of DMF, to which DIEA (4 equiv) was added. The amino acid was preactivated for 1 min, then the solution was added to the resin. After agitated for 6h, the resin was washed with DMF (× 3), DCM (× 3) and DMF (× 3), the coupling cycle was repeated for 2 times.

9-[(9H-Fluorenylmethoxy) carbonyl amino]-4,7-dioxanonanoic Acid (1.2 equiv), HATU (1.2 equiv) was dissolved in 4 mL of DMF, to which DIEA (2.4 equiv) was added. The amino acid was preactivated for 1 min, then the solution was added to the resin. After agitated for 16h, the resin was washed with DMF (× 3), DCM (× 3) and DMF (× 3).

6-[(9H-Fluorenylmethoxy) carbonyl amino] hexanoic acid (4 equiv), HATU (4 equiv) was dissolved in 4 mL of DMF, to which DIEA (8 equiv) was added. The amino acid was preactivated for 1 min, then the solution was added to the resin. After agitated for 20min, the resin was washed with DMF (× 3), DCM (× 3) and DMF (× 3).

The 2-chlorotritylchloride resin (0.970 mmol/g) employed in SPPS was purchased from GL Biochem.

3.3 Preparation of Peptidyl Acids and N-Acetyl Capped Peptidyl Acids

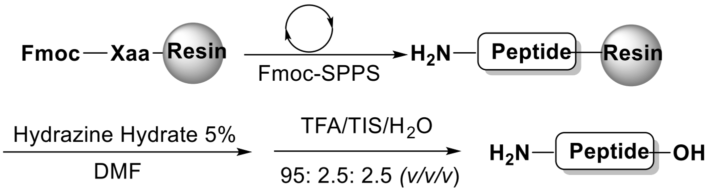

Scheme S4. Preparation of peptidyl acids.

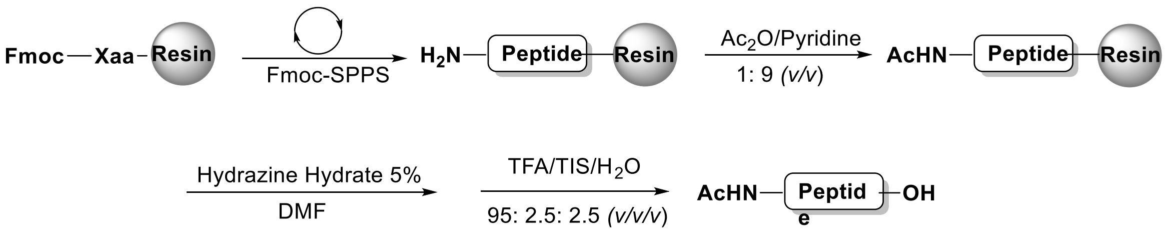

Scheme S5. Preparation of acetyl capped peptidyl acids.

Upon completion of the automated synthesis on a 0.05 mmol scale, the peptide resin was washed into a peptide synthesis vessel using DCM. The acetyl groups on the monosaccharides was removed by 5% hydrazine hydrate in DMF for 16 h. Resin cleavage and global deprotection was performed under the treatment of TFA/TIS/H_2O (95:2.5:2.5, *v/v/v*) solution for 2 hours (Scheme S4). The resin was then removed by filtration, and the filtrate was concentrated under a nitrogen atmosphere. The resulting residue was triturated with cold diethyl ether to give a white solid, which was then dissolved in a solution of MeCN and water containing 5% of acetic acid. The resulting solution was ready for HPLC purification after filtration.

The acetyl capped peptidyl acids could be prepared by adding a step using Ac_2O and pyridine (1:9, *v/v*) before the treatment of hydrazine hydrate (Scheme S5). Resin cleavage, global deprotection and further treatment was performed under the same steps.

IV. Preparation and Characterization of Peptide Sequence

Peptide conjugate 4

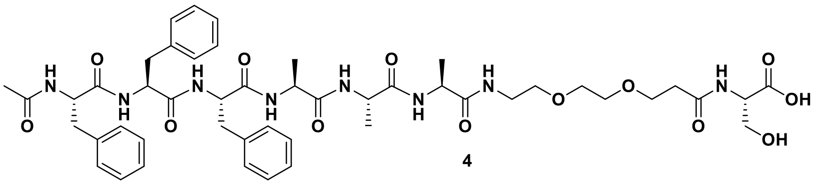

Chemical Formula: $C_{48}H_{64}N_8O_{13}$
Exact Mass: 960.46
Molecular Weight: 961.08

Compound **4** was prepared according to procedures **3.2** and **3.3** described above on a 0.05 mmol scale. Purification of the crude peptide using preparative HPLC (10 to 70% solvent B over 30 min, Agilent Eclipse XDB-C18 column) afforded compound **4** as a white solid after lyophilization (9.6 mg, 20%).

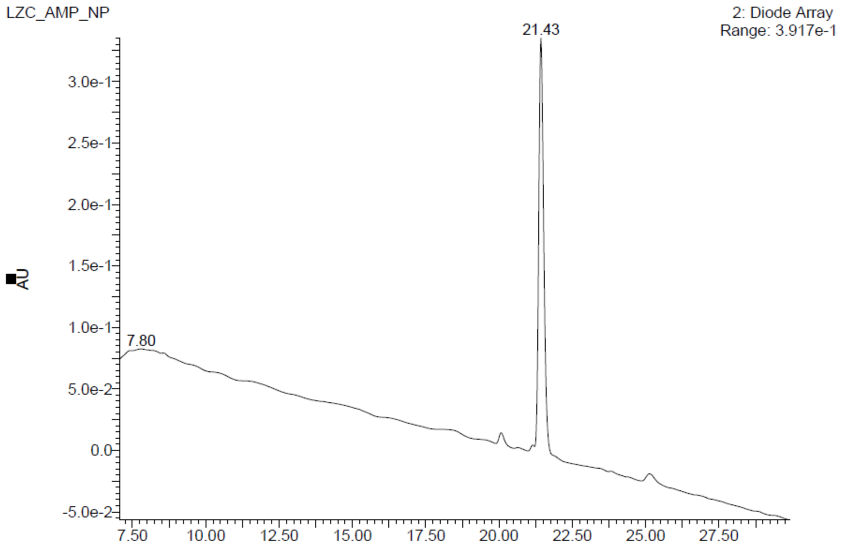

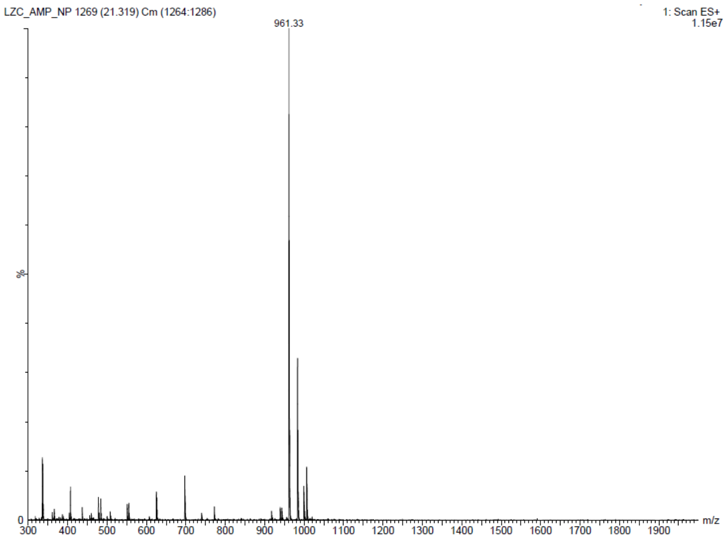

Figure S1. Top: UV trace of the compound **4**. Linear gradient: 10 to 70% solvent B over 30 min, Agilent C18 column, t_R = 21.43 min; Bottom: ESI-MS data of the purified compound **4** (m/z): $[M+H]^+$ Calcd for $C_{48}H_{65}N_8O_{13}$, 962.09 Da (average isotopes); found, 961.33.

Peptide conjugate 5

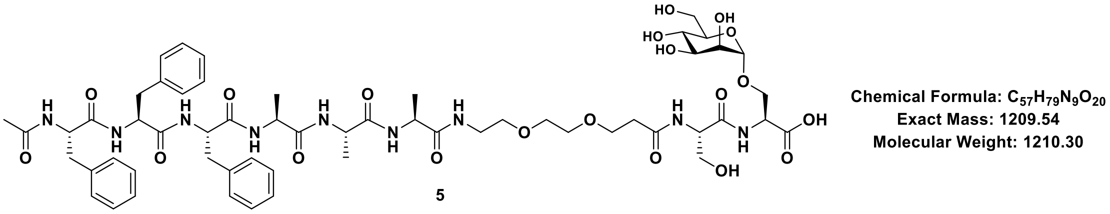

5

Compound **5** was prepared according to procedures **3.2** and **3.3** described above on a 0.05 mmol scale. Purification of the crude peptide using preparative HPLC (10 to 70% solvent B over 30 min, Agilent Eclipse XDB-C18 column) afforded compound **5** as a white solid after lyophilization (11 mg, 18%).

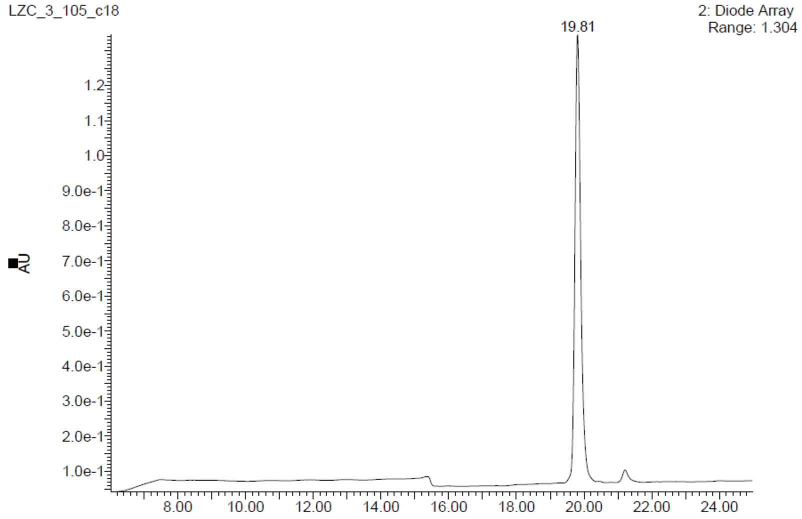

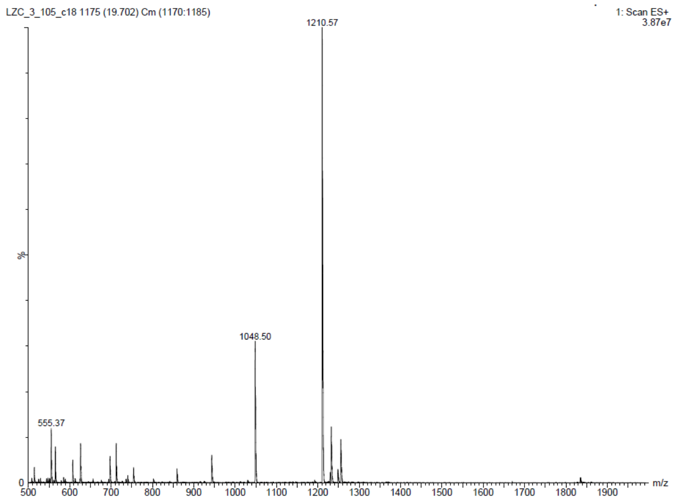

Figure S2. Top: UV trace of the compound **5**. Linear gradient: 10 to 70% solvent B over 30 min, Agilent C18 column, t_R = 19.81 min; Bottom: ESI-MS data of the purified compound **5** (m/z): $[M+H]^+$ Calcd for $C_{57}H_{80}N_9O_{20}$, 1211.31 Da (average isotopes); found, 1210.57.

Peptide conjugate 6 (Shell-NPs)

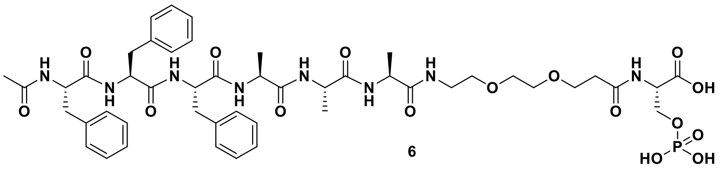

Chemical Formula: $C_{48}H_{65}N_8O_{16}P$
Exact Mass: 1040.43
Molecular Weight: 1041.06

Compound **6** was prepared according to procedures **3.2** and **3.3** described above on a 0.05 mmol scale. Purification of the crude peptide using preparative HPLC (5 to 50% solvent B over 30 min, Agilent Eclipse XDB-C18 column) afforded compound **6** as a white solid after lyophilization (5 mg, 10%).

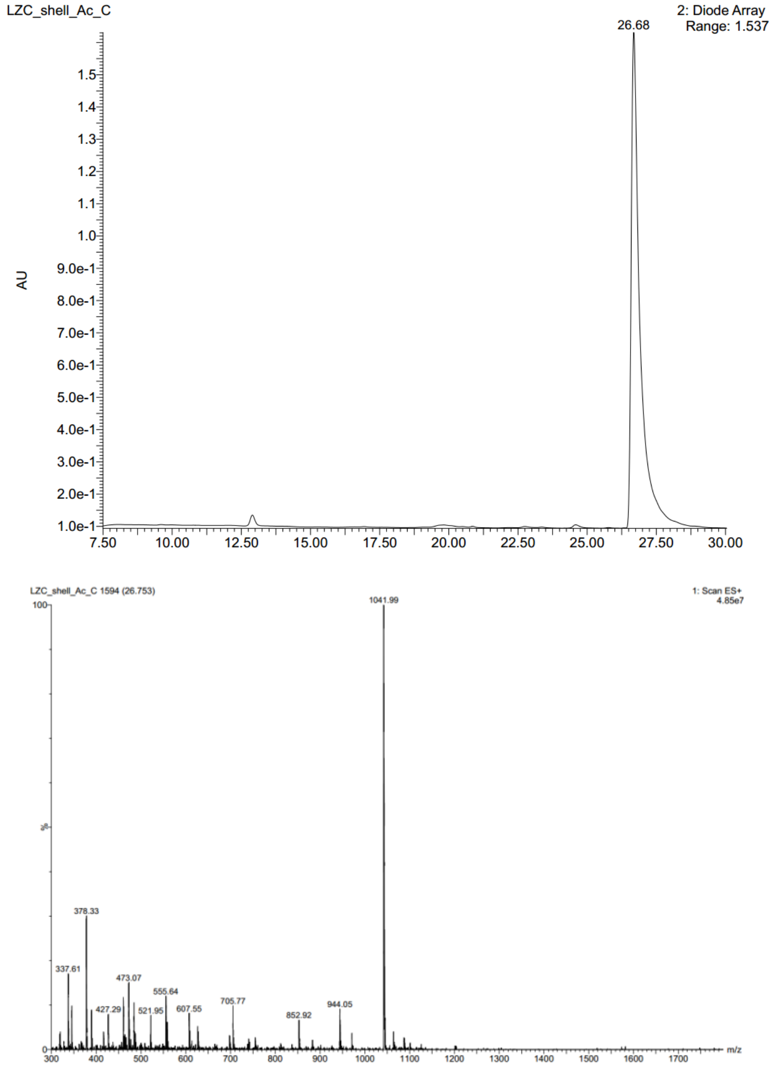

Figure S3. Top: UV trace of the compound **6**. Linear gradient: 5 to 50% solvent B over 30 min, Agilent C18 column, t_R = 26.68 min; Bottom: ESI-MS data of the purified compound **6** (*m/z*): $[M+H]^+$ Calcd for $C_{48}H_{66}N_8O_{16}P$: 1042.07 Da (average isotopes); found, 1041.90.

Peptide conjugate 8

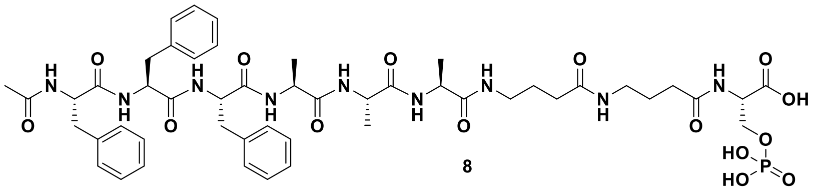

Chemical Formula: $C_{49}H_{66}N_9O_{15}P$
Exact Mass: 1051.44
Molecular Weight: 1052.09

Compound **8** was prepared according to procedures **3.2** and **3.3** described above on a 0.05 mmol scale. Purification of the crude peptide using preparative HPLC (10 to 70% solvent B over 30 min, Agilent Eclipse XDB-C18 column) afforded compound **8** as a white solid after lyophilization (2 mg, 4%).

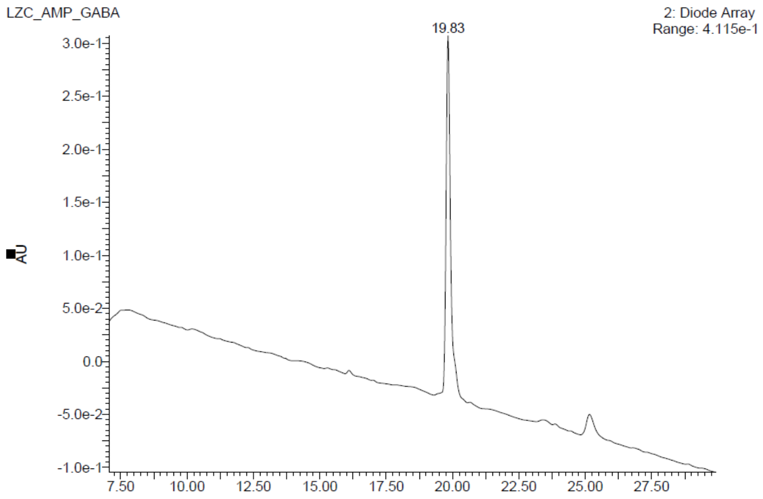

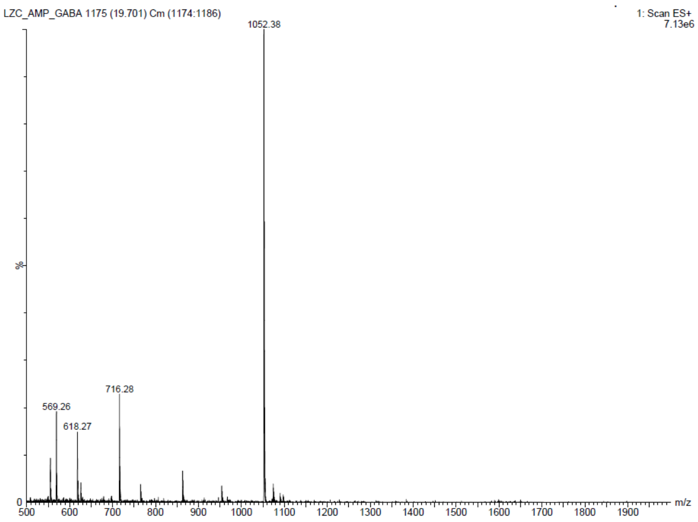

Figure S4. Top: UV trace of the compound **8**. Linear gradient: 10 to 70% solvent B over 30 min, Agilent C18 column, t_R = 19.83 min; Bottom: ESI-MS data of the purified compound **8** (m/z): $[M+H]^+$ Calcd for $C_{49}H_{67}N_9O_{15}P$, 1053.10 Da (average isotopes); found, 1052.38.

Peptide conjugate 9

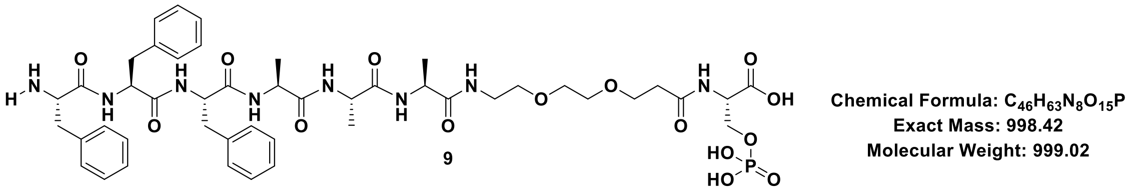

Compound **9** was prepared according to procedures **3.2** and **3.3** described above on a 0.05 mmol scale. Purification of the crude peptide using preparative HPLC (10 to 70% solvent B over 30 min, Agilent Eclipse XDB-C18 column) afforded compound **9** as a white solid after lyophilization (4 mg, 8%).

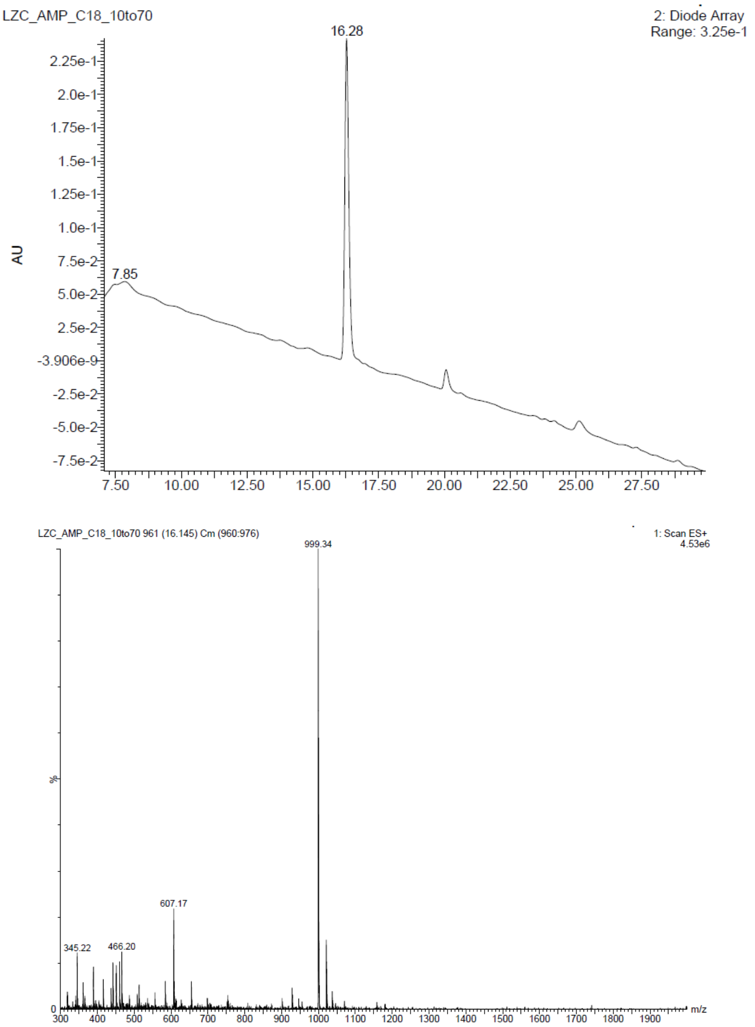

Figure S5. Top: UV trace of the compound **9**. Linear gradient: 10 to 70% solvent B over 30 min, Agilent C18 column, t_R = 16.28 min; Bottom: ESI-MS data of the purified compound **9** (m/z): $[M+H]^+$ Calcd for $C_{46}H_{64}N_8O_{15}P$, 1000.03 Da (average isotopes); found, 999.34.

Peptide conjugate 10 (Man-NPs)

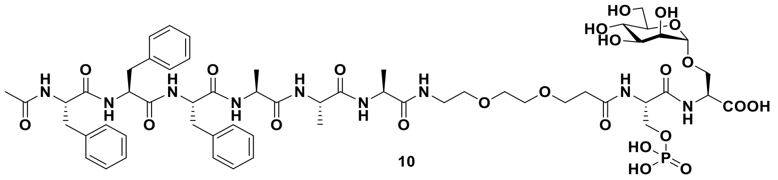

Compound **10** was prepared according to procedures **3.2** and **3.3** described above on a 0.05 mmol scale. Purification of the crude peptide using preparative HPLC (5 to 50% solvent B over 30 min, Agilent Eclipse XDB-C18 column) afforded compound **10** as a white solid after lyophilization (18 mg, 28%).

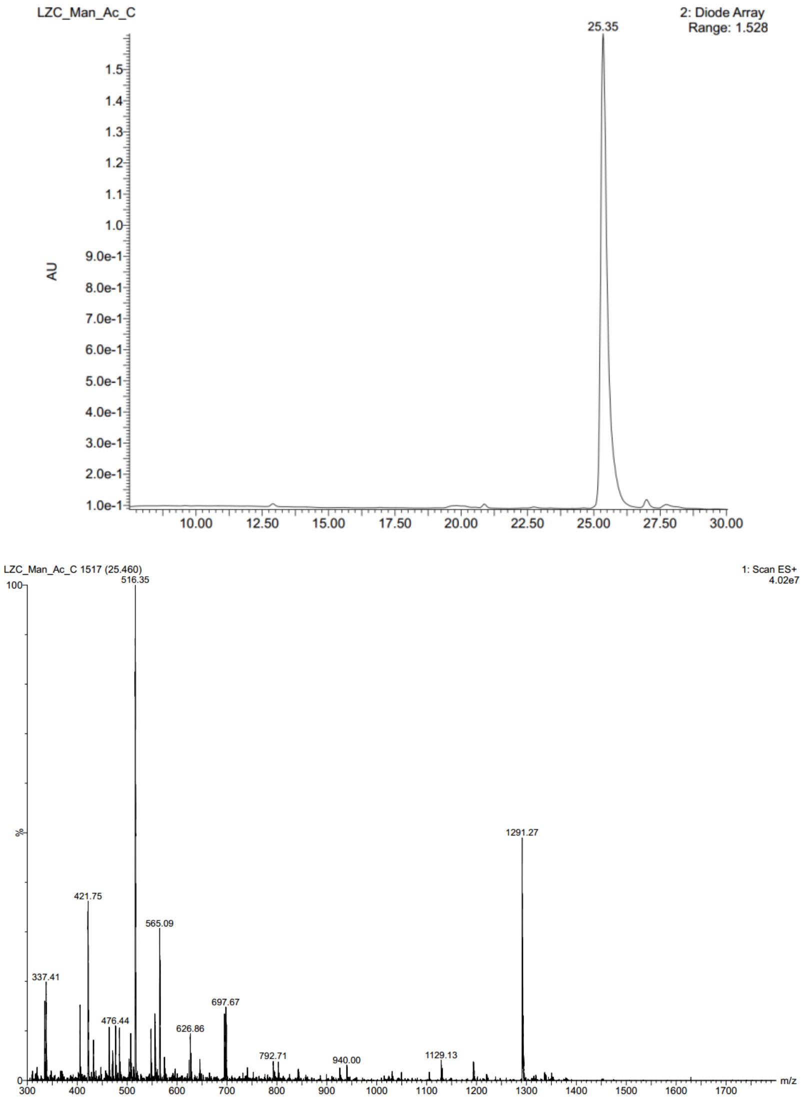

Figure S6. Top: UV trace of the compound **10**. Linear gradient: 5 to 50% solvent B over 30 min, Agilent C18 column, t_R = 25.35 min; Bottom: ESI-MS data of the purified compound **10** (m/z): $[M+H]^+$ Calcd for $C_{57}H_{81}N_8O_{23}P$, 1291.29 Da (average isotopes); found, 1291.27.

Peptide conjugate 11 (Gal-NPs)

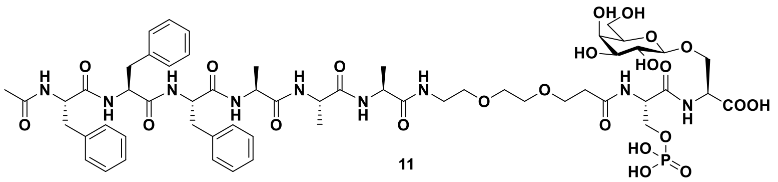

Compound **11** was prepared according to procedures **3.2** and **3.3** described above on a 0.05 mmol scale. Purification of the crude peptide using preparative HPLC (5 to 50% solvent B over 30 min, Agilent Eclipse XDB-C18 column) afforded compound **11** as a white solid after lyophilization (8 mg, 12%).

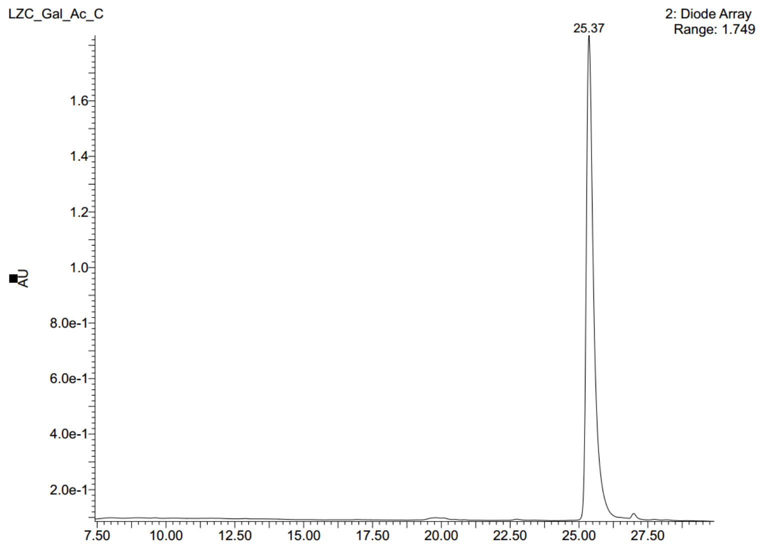

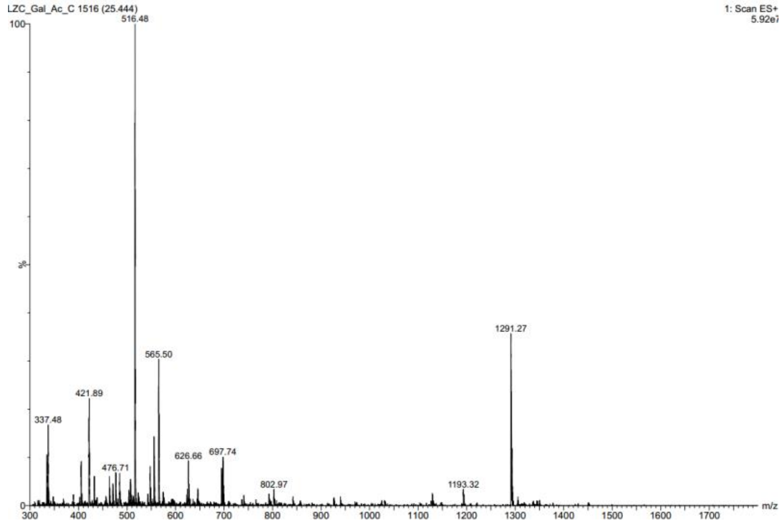

Figure S7. Top: UV trace of the compound **11**. Linear gradient: 5 to 50% solvent B over 30 min, Agilent C18 column, t_R = 25.37 min; Bottom: ESI-MS data of the purified compound **11** (m/z): $[M+H]^+$ Calcd for $C_{57}H_{81}N_8O_{23}P$, 1291.29 Da (average isotopes); found, 1291.27.

Peptide conjugate 12 (Glc-NPs)

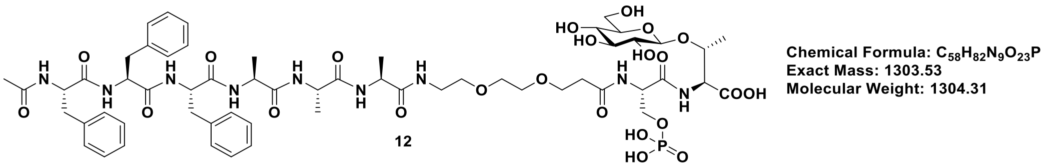

Compound **12** was prepared according to procedures **3.2** and **3.3** described above on a 0.05 mmol scale. Purification of the crude peptide using preparative HPLC (5 to 50% solvent B over 30 min, Agilent Eclipse XDB-C18 column) afforded compound **12** as a white solid after lyophilization (8 mg, 12%).

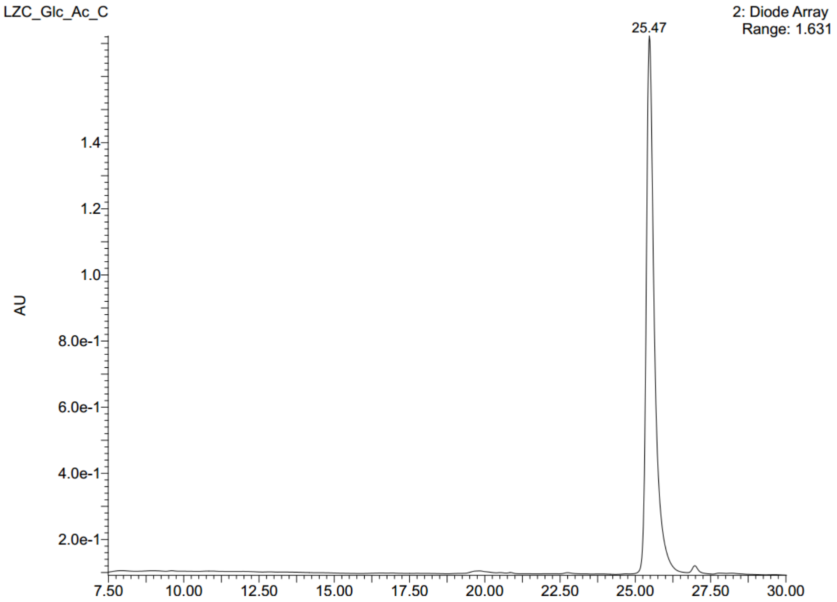

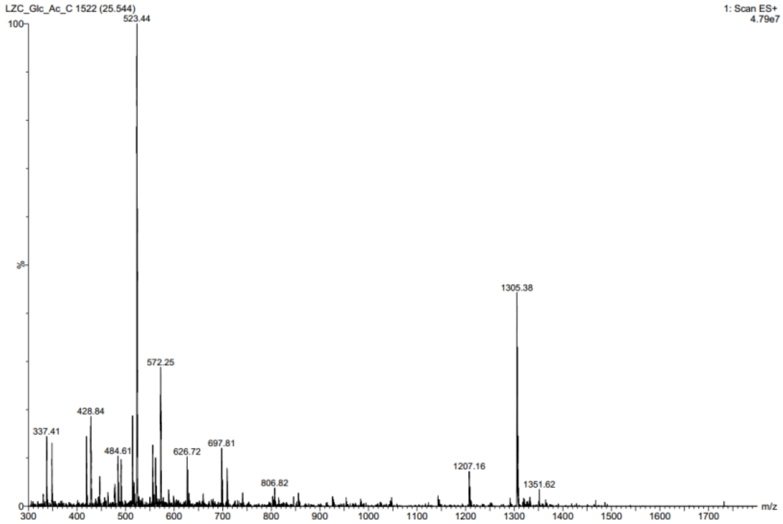

Figure S8. Top: UV trace of the compound **12**. Linear gradient: 5 to 50% solvent B over 30 min, Agilent C18 column, t_R = 25.47 min; Bottom: ESI-MS data of the purified compound **12** (m/z): $[M+H]^+$ Calcd for $C_{58}H_{83}N_9O_{23}P$, 1305.32 Da (average isotopes); found, 1305.38.

Peptide conjugate 13

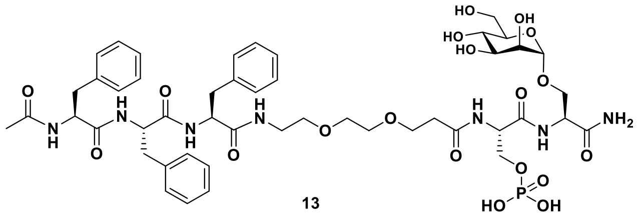

13

Chemical Formula: $C_{48}H_{66}N_7O_{19}P$
Exact Mass: 1075.42
Molecular Weight: 1076.06

Compound **13** was prepared according to procedures **3.2** and **3.3** described above on a 0.05 mmol scale. Purification of the crude peptide using preparative HPLC (10 to 70% solvent B over 30 min, Agilent Eclipse XDB-C18 column) afforded compound **13** as a white solid after lyophilization (9 mg, 16%).

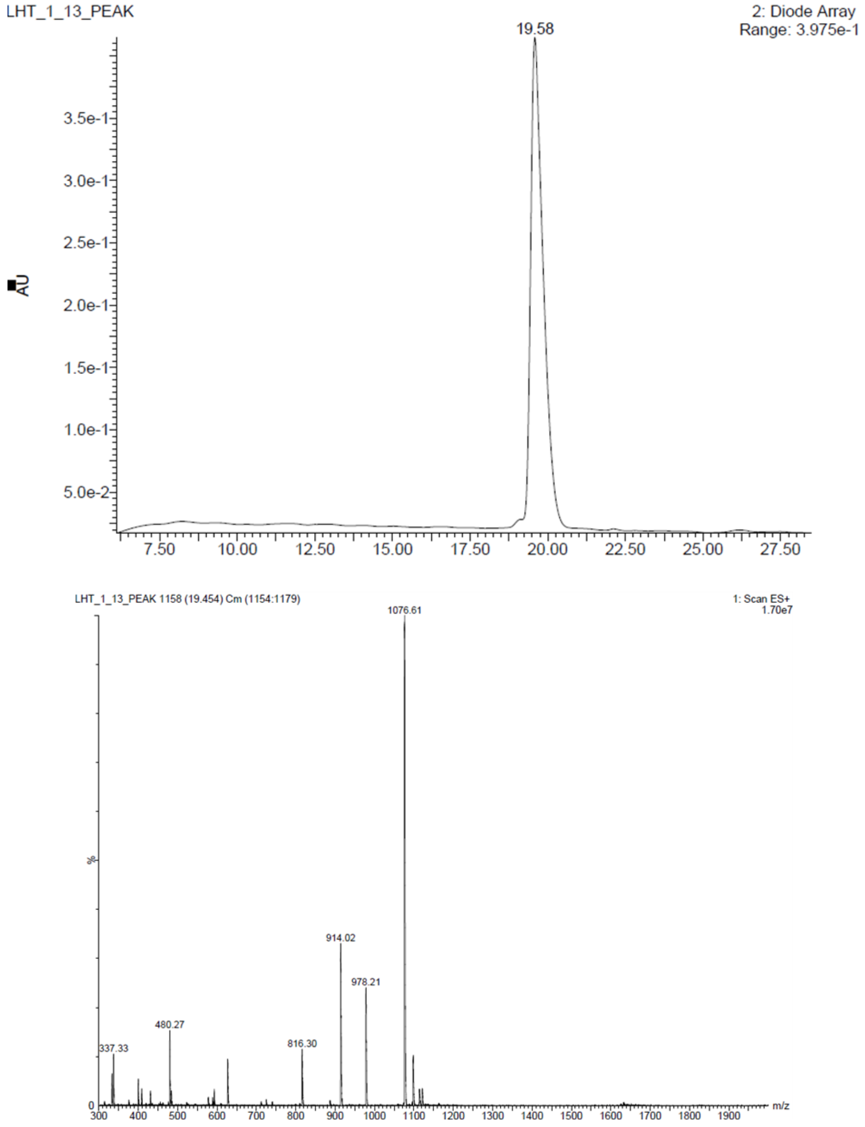

Figure S9. Top: UV trace of the compound **13**. Linear gradient: 10 to 70% solvent B over 30 min, Agilent C18 column, t_R = 19.58 min; Bottom: ESI-MS data of the purified compound **13** (m/z): $[M+H]^+$ Calcd for $C_{48}H_{67}N_7O_{19}P$, 1077.07 Da (average isotopes); found, 1076.61.

V. Preparation of FITC-Labeled Nanoparticles

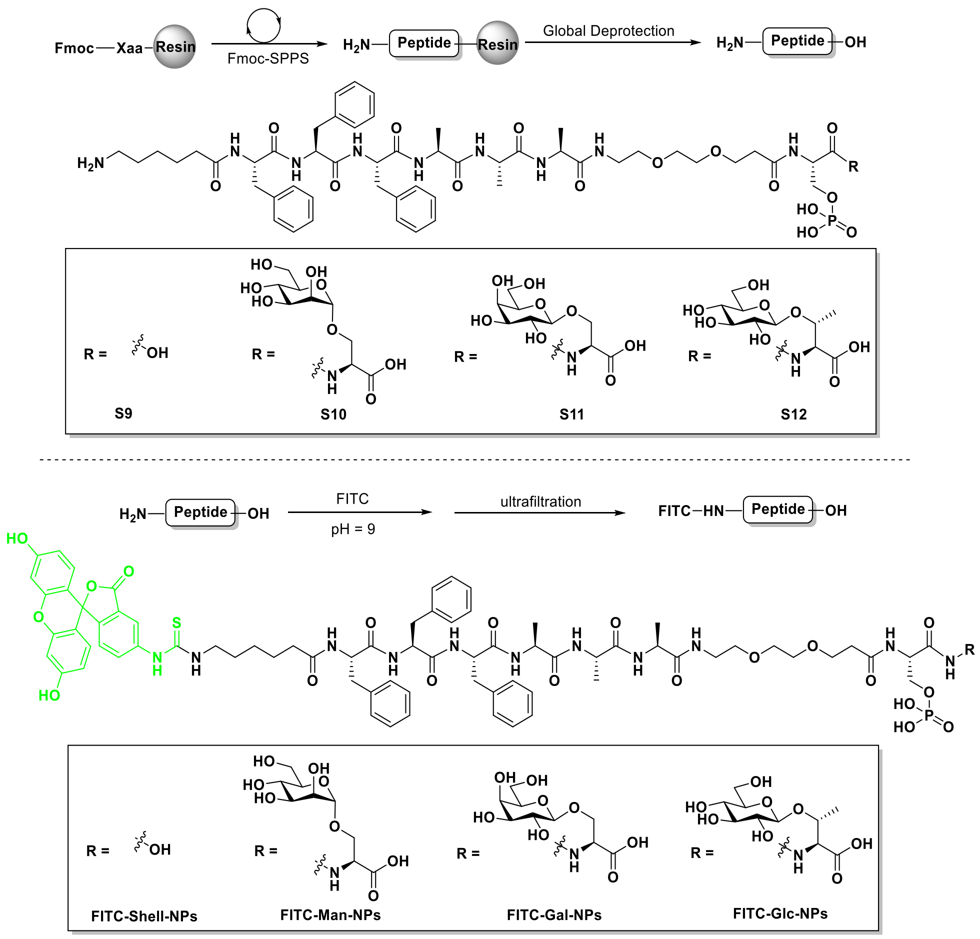

Scheme S6. Preparation of FITC-labeled nanoparticles

5.1 Preparation of Aminocaproic Acid Linked Peptidyl Acids

Peptide conjugate S9

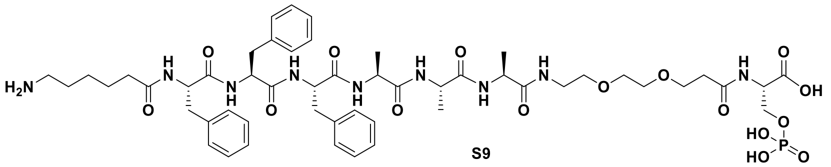

S9

Chemical Formula: $C_{52}H_{74}N_9O_{16}P$
Exact Mass: 1111.50
Molecular Weight: 1112.18

Compound **S9** was prepared according to procedures **3.2** and **3.3** described above on a 0.05 mmol scale. Purification of the crude peptide using preparative HPLC (5 to 50% solvent B over 30 min, Agilent Eclipse XDB-C18 column) afforded compound **S9** as a white solid after lyophilization (12 mg, 22%).

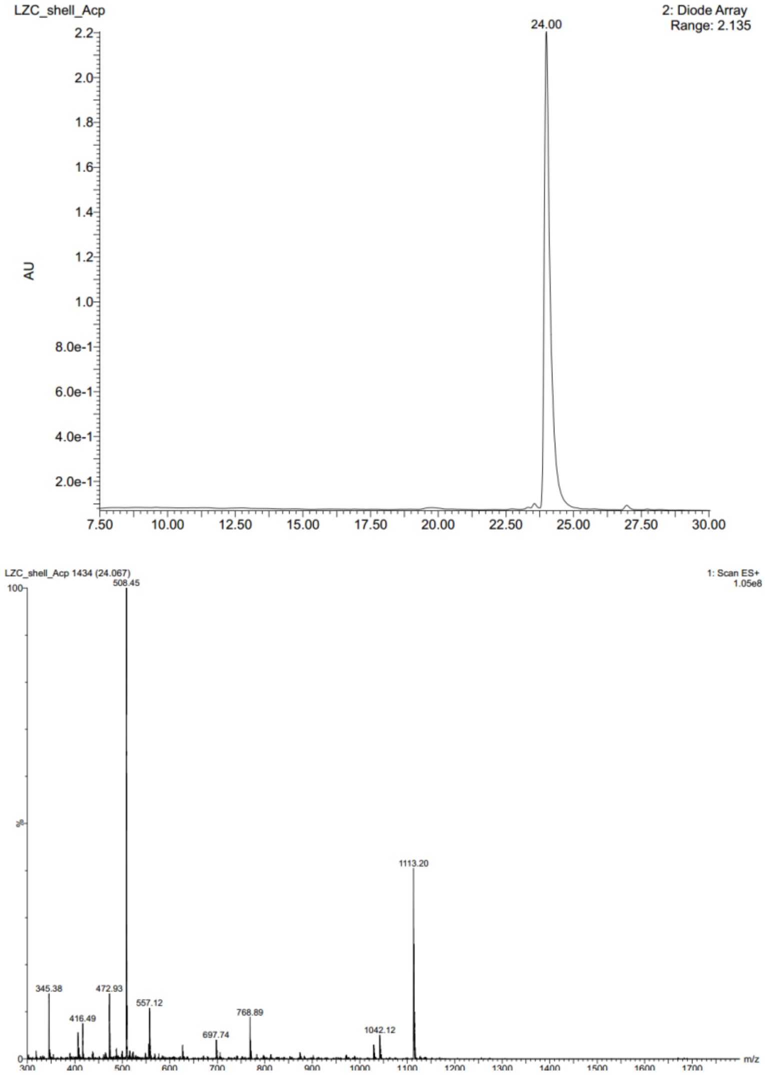

Figure S10. Top: UV trace of the compound **S9**. Linear gradient: 5 to 50% solvent B over 30 min, Agilent C18 column, t_R = 24.00 min; ESI-MS data of the purified compound **S9** (m/z): $[M+H]^+$ Calcd for $C_{52}H_{75}N_9O_{16}P$, 1113.19 Da (average isotopes); found, 1113.20.

Peptide conjugate S10

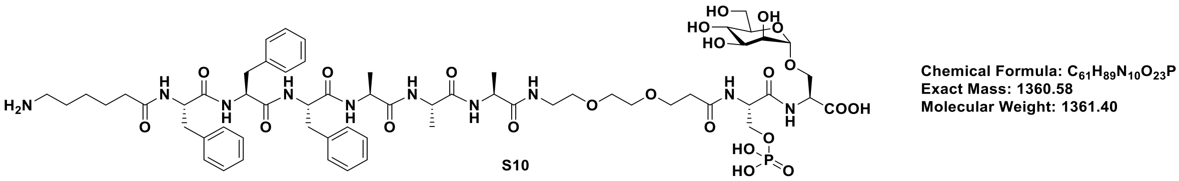

S10

Compound **S10** was prepared according to procedures **3.2** and **3.3** described above on a 0.05 mmol scale. Purification of the crude peptide using preparative HPLC (5 to 50% solvent B over 30 min, Agilent Eclipse XDB-C18 column) afforded compound **S10** as a white solid after lyophilization (20 mg, 29%).

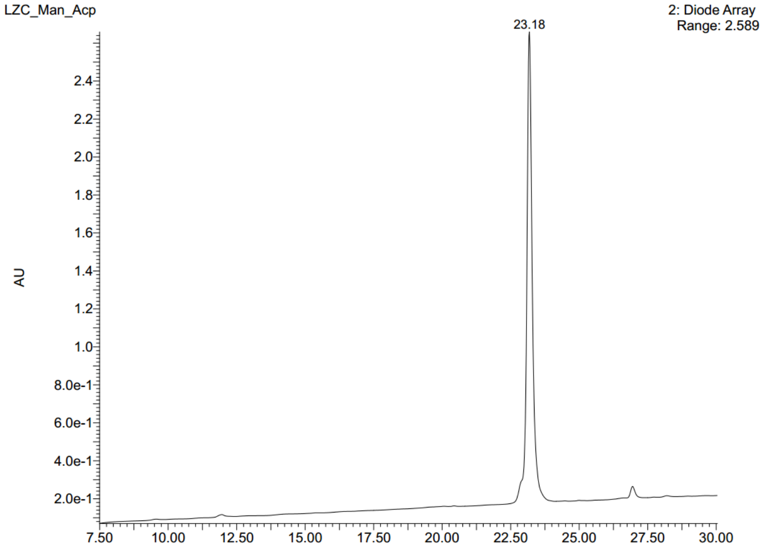

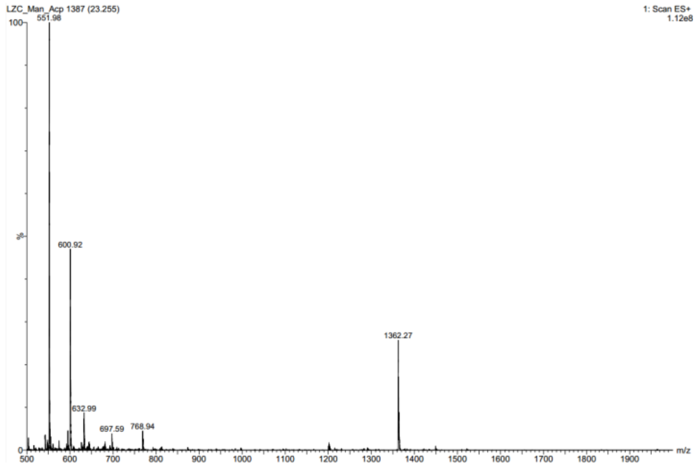

Figure S11. Top: UV trace of the compound **S10**. Linear gradient: 5 to 50% solvent B over 30 min, Agilent C18 column, t_R = 23.18 min; Bottom: ESI-MS data of the purified compound **S10** (m/z): $[M+H]^+$ Calcd for $C_{61}H_{90}N_{10}O_{23}P$, 1362.41 Da (average isotopes); found, 1362.27.

Peptide conjugate S11

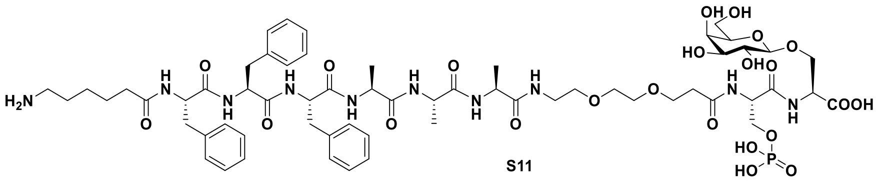

S11

Chemical Formula: $C_{61}H_{89}N_{10}O_{23}P$
Exact Mass: 1360.58
Molecular Weight: 1361.40

Compound **S11** was prepared according to procedures **3.2** and **3.3** described above on a 0.05 mmol scale. Purification of the crude peptide using preparative HPLC (5 to 50% solvent B over 30 min, Agilent Eclipse XDB-C18 column) afforded compound **S11** as a white solid after lyophilization (14 mg, 21%).

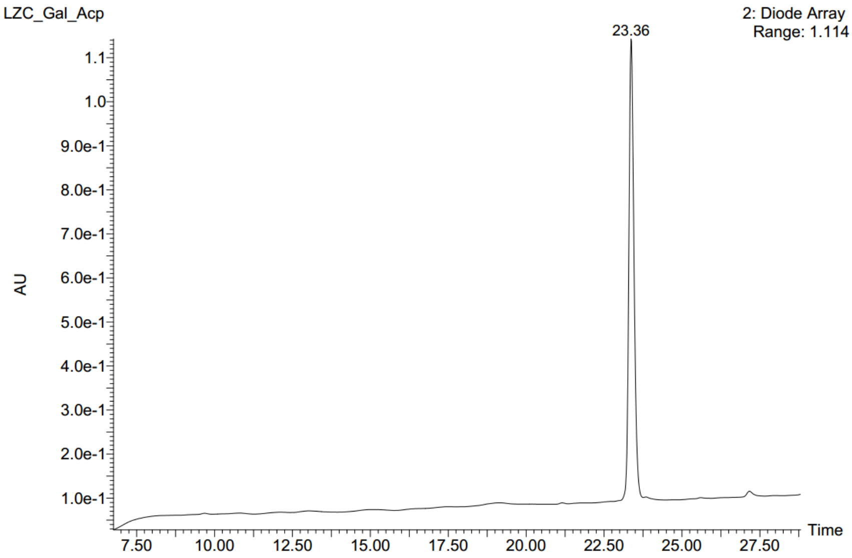

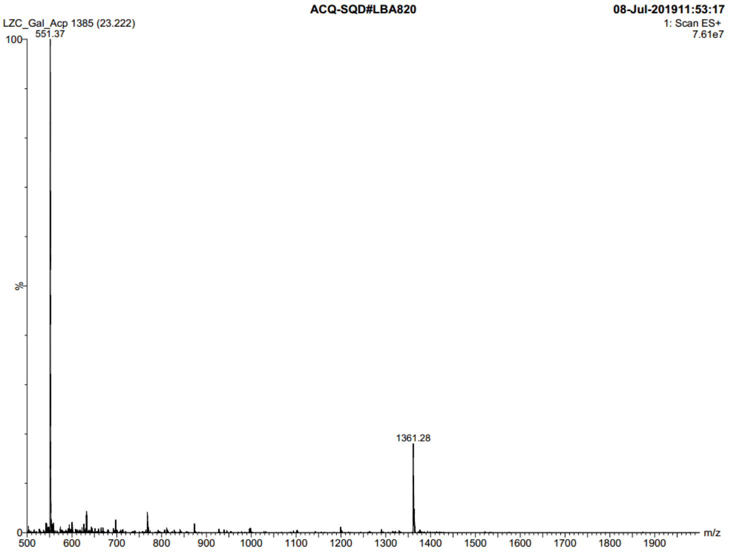

Figure S12. Top: UV trace of the compound **S11**. Linear gradient: 5 to 50% solvent B over 30 min, Agilent C18 column, t_R = 23.36 min; Bottom: ESI-MS data of the purified compound **S11** (m/z): $[M+H]^+$ Calcd for $C_{61}H_{90}N_{10}O_{23}P$, 1362.41 Da (average isotopes); found, 1361.28.

Peptide conjugate S12

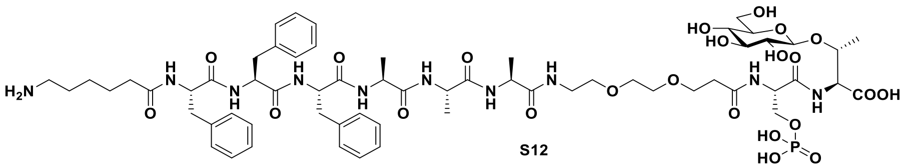

S12

Chemical Formula: $C_{62}H_{91}N_{10}O_{23}P$
Exact Mass: 1374.60
Molecular Weight: 1375.43

Compound **S12** was prepared according to procedure **3.2** and **3.3** describe above on a 0.05 mmol scale. Purification of the crude peptide using preparative HPLC (5 to 50% solvent B over 30 min, Agilent Eclipse XDB-C18 column) afforded compound **S12** as a white solid after lyophilization (14 mg, 20%).

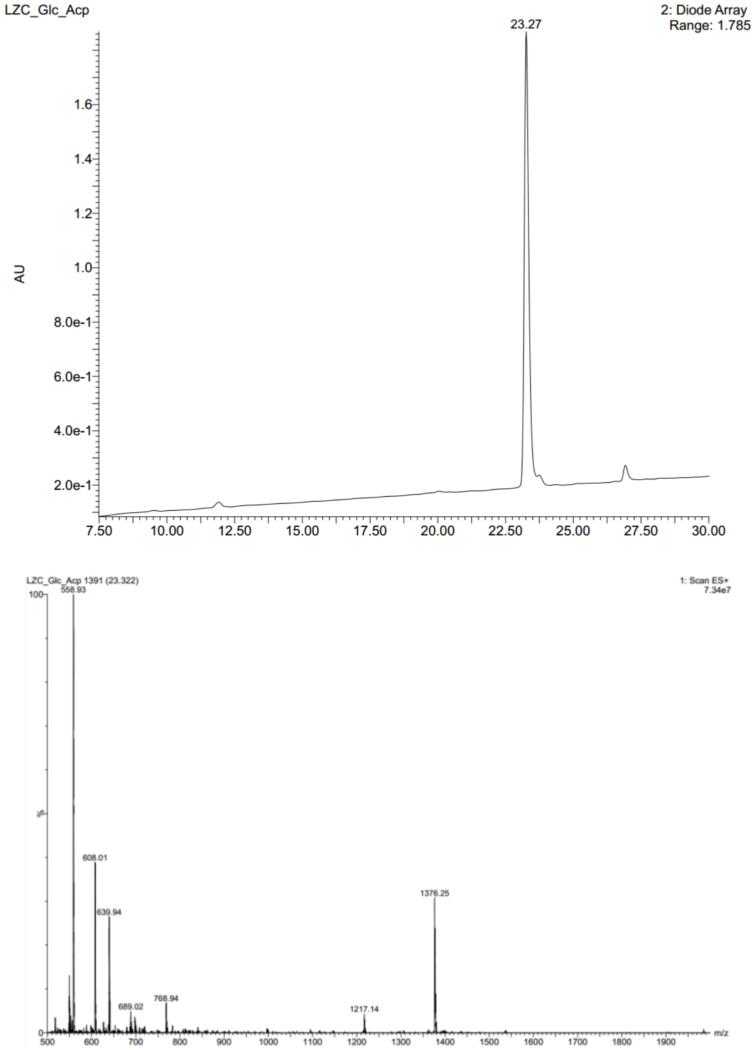

Figure S13. Top: UV trace of the compound **S12**. Linear gradient: 5 to 50% solvent B over 30 min, Agilent C18 column, t_R = 23.27 min; Bottom: ESI-MS data of the purified compound **S12** (m/z): $[M+H]^+$ Calcd for $C_{62}H_{92}N_{10}O_{23}P$, 1376.44 Da (average isotopes); found, 1376.25.

5.2 Representative data of DLS experiments on FITC-Labeled Nanoparticles

Peptide conjugates **S9**, **S10**, **S11** and **S12** were prepared and purified according to procedures described in the manuscript.

DLS experiments indicate that the **S10**-derived FITC-labeled conjugate could also form uniform nanoparticles similar to that from peptide conjugate **6**, as shown in Figure S14, suggesting that FITC-labeling did not compromise the self-assembling or affect the morphology of generated NPs.

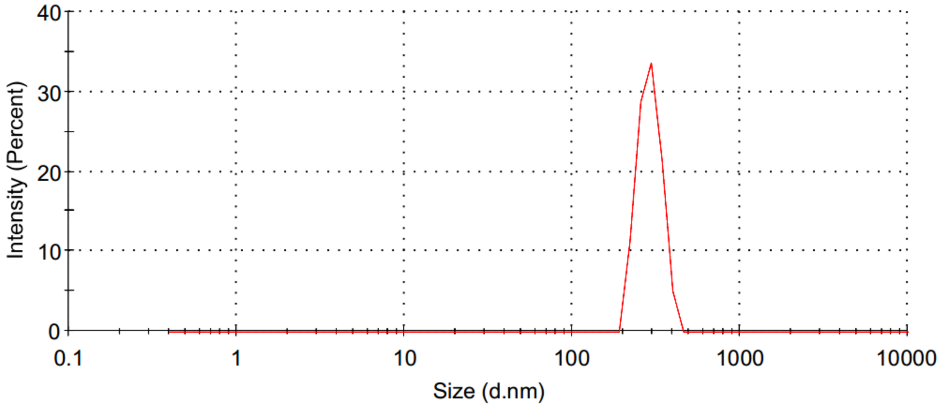

Figure S14. Intensity size distribution of FITC labeled nanoparticles.

VI. Characterization of Nano Particulate

6.1 Dynamic Light Scattering and Zeta Potential Analysis

ZETASIZER NANO ZSP was employed to perform the dynamic light scattering experiment. Data was listed in table **S1**.

Table S1. Data from the DLS experiments.

Construct	*Diameter (nm)*	*PDI*	*Zeta Potential*
Shell-NPs	405.0	0.464	-12.9
Man-NPs	435.2	0.628	-32.2
Gal-NPs	520.1	0.554	-35.1
Glc-NPs	511.2	0.537	-28.8

6.2 Transmission Electron Microscopy Analysis

20 μL solution of different sequences (100 μg/ml) was added dropwise on the copper grids of carbon support films for 1min 20 second respectively. Then 20 μL phosphotungstic acid was added for 1min. After dried, copper grids were imaged on transmission electron microscopy (JEM1200EX transmission electron microscope). Besides the TEM pictures in manuscript, the TEM pictures of entry 6 and entry 10 were reported in figure S15 and figure S16.

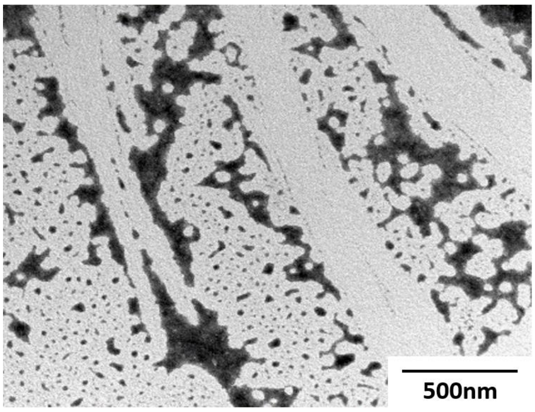

Figure S15. Serious aggregation of peptide **13** (entry 6).

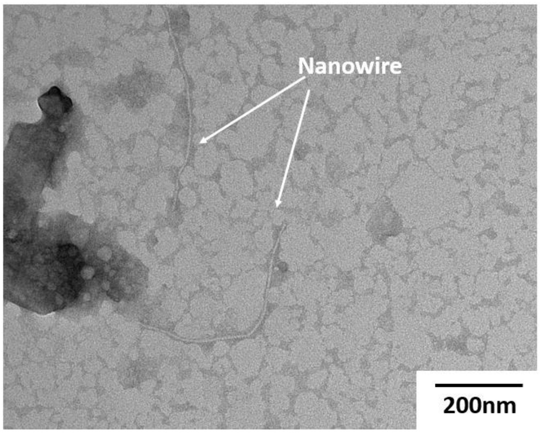

Figure S16. Aggregation and formed nanowire of peptide **17** (entry 10)

VII. *In Vitro* Study of Glycosylated Nanoparticles

7.1 Blocking MMR with anti-MMR Antibody

RAW 264.7 cells were diluted to 1×10^5 cells/mL and 1 mL media was added to each well (1×10^5 cells) in 12-well plate. Mixture of FITC-Man-NPs and anti-MMR antibody was added as experimental group while the control group was added FITC-Man-NPs only. After incubation for 24 hours at 37 ℃, the media was removed. After washing with DPBS, fixed with 4% paraformaldehyde for 20 min, the samples were observed by confocal microscopy in FITC channels and bright field.

7.2 SPR Experiments and Data

The interaction between ConA protein and Man-NPs as well as unmodified mannose was measured on Biacore 8k (GE Healthcare) instrument at 25 ℃ in a running buffer containing PBS, and 0.05% surfactant P20. ConA protein (Solarbio) was immobilized on a sensor chip (CM5) with a final level about 10000RU, the results were reported in Figure S17 and Figure S18. No regeneration of the chip surface was required between different analyte injections.

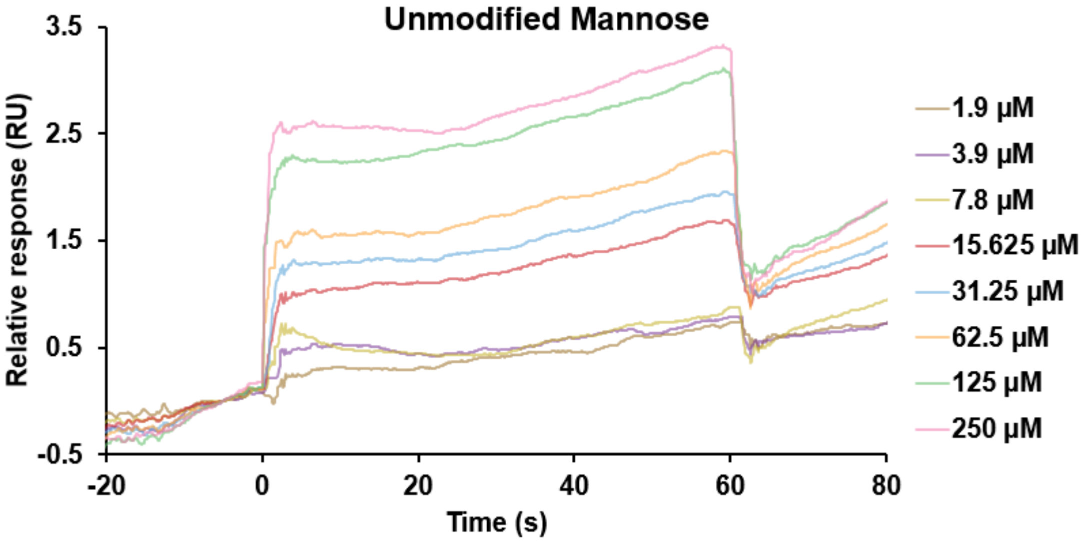

Figure S17. SPR analysis of interaction of unmodified mannose and ConA protein

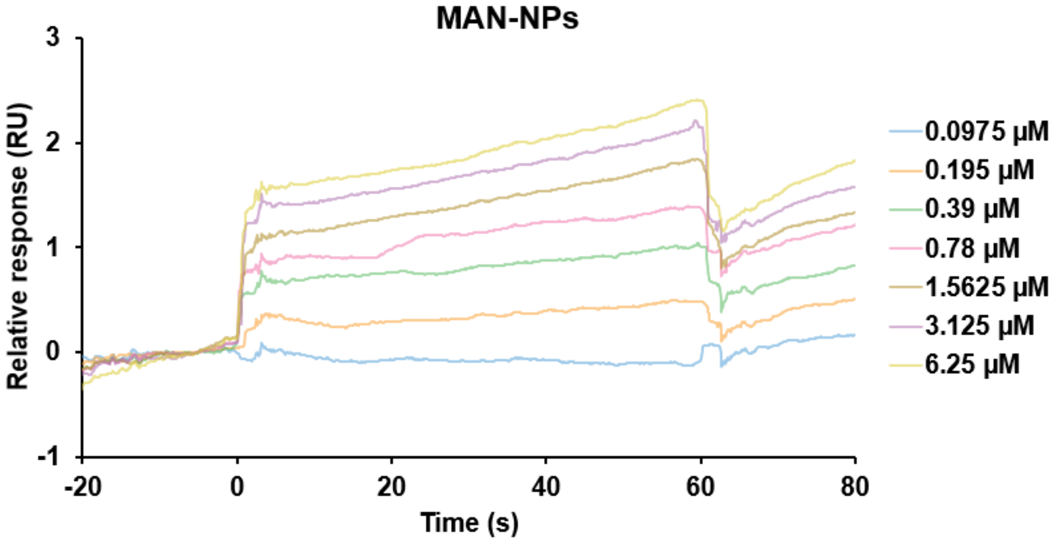

Figure S18. SPR analysis of interaction of Man-NPs and ConA protein
